# CD-73 blocking black phosphorus nanosheets hitchhiked on erythrocytes *via* affinity difference of AS1411 aptamer for targeted photothermal-immunotherapy of lung cancer

**DOI:** 10.1016/j.mtbio.2026.103559

**Published:** 2026-08-13

**Authors:** Qiao Liu, Niu Liu, Zhiqi Lao, Jian Qin, Yuting Zhong, Kaiwei Hou, Yuetao Zhao

**Affiliations:** a Department of Biochemistry and Molecular Biology, School of Life Sciences, Central South University, No.172 Tongzipo Road, Changsha, 410013, China; b Institute of Biomedicine and Biotechnology, Shenzhen Institute of Advanced Technology, Chinese Academy of Sciences, Shenzhen, China; c Changsha Stomatological Hospital, School of Stomatology, Hunan University of Chinese Medicine, Changsha, Hunan, China

**Keywords:** Biomimetic nanomaterials, Erythrocytes-hitchhiking, Black phosphorus, Tumor combined therapy, Photothermal-immunotherapy

## Abstract

Metabolic checkpoint inhibition targeting adenosine signaling combined with photothermal therapy (PTT) can remodel the immunosuppressive tumor microenvironment and enhance antitumor immunity in lung cancer, but its efficacy is limited by tumor-targeted accumulation. Leveraging the differential binding affinity of AS1411 aptamer (Apt) for nucleolin and actin, we developed an erythrocytes-based nanodelivery system incorporating red blood cell (RBC), black phosphorus nanosheets (BPs), the CD73 inhibitor AMPCP, and Apt. The Apt binds to actin on RBC membranes, facilitating BPs and AMPCP loading and enabling RBC hitchhiking to prolong systemic circulation and enhance tumor accumulation. At the tumor site, the higher affinity for nucleolin of aptamer triggers the release of BPs and AMPCP, which combinedly increase dendritic cell maturation and expand CD8^+^ T cells, while reducing regulatory T cells and myeloid-derived suppressor cells. The combined PTT and CD-73 blockade reshapes the immunosuppressive microenvironment and boosts potential antitumor immunity, significantly suppresses lung cancer growth. This RBC-based delivery strategy facilitates targeted nanomedicine accumulation and robust antitumor immune activation, providing an effective platform for lung cancer suppression.

## Graphical abstract

**Figure:**
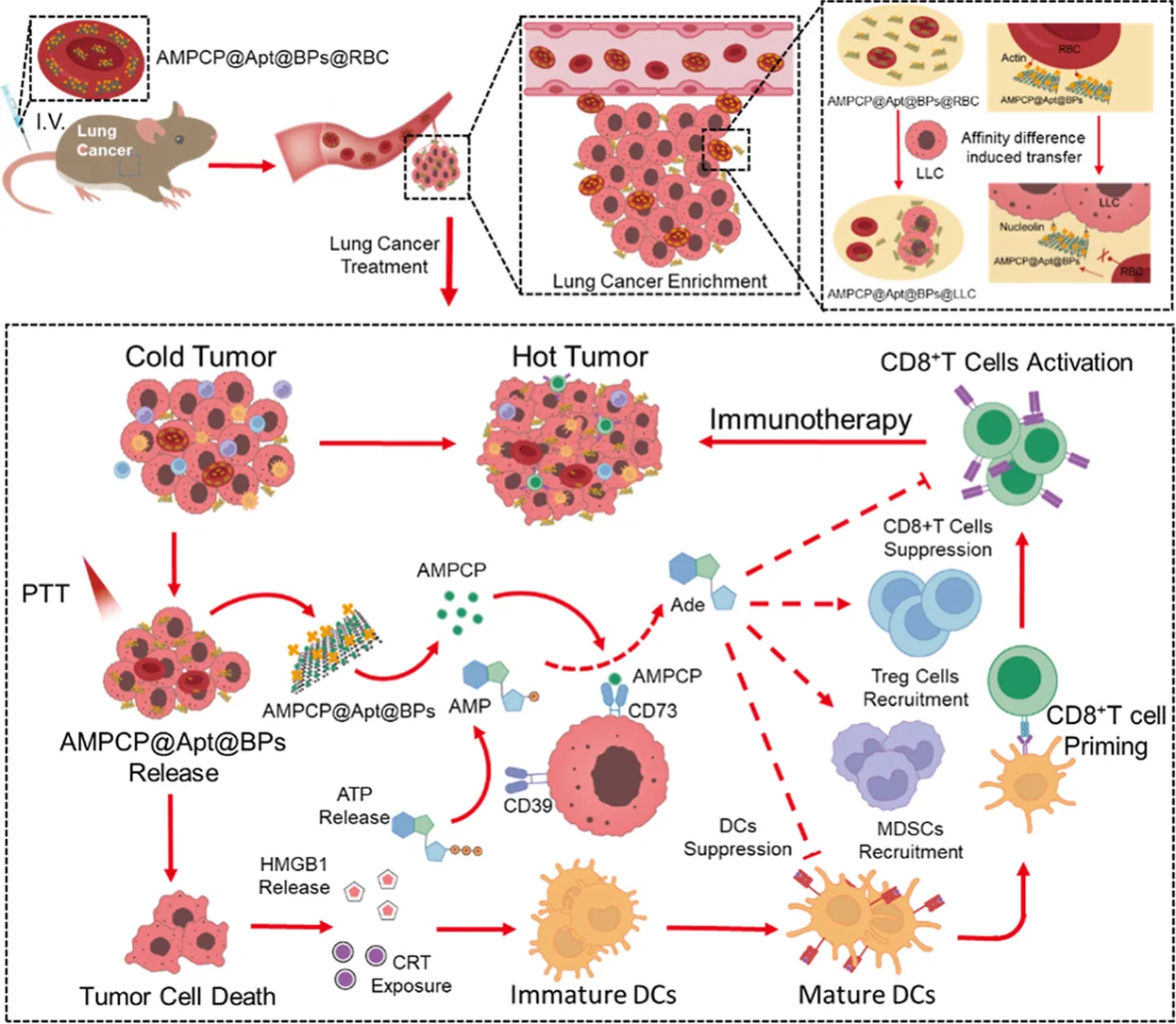

## 1 Introduction

Lung cancer exhibits a high incidence rate worldwide and ranks as the leading cause of cancer-related mortality. According to estimates by the International Agency for Research on Cancer (IARC), lung cancer results in approximately 1.8 million deaths in 2020 [1]. Immune checkpoint inhibitors (ICIs) targeting PD-1/PD-L1 and CTLA-4 play an increasingly significant role in the clinical management of non-small cell lung cancer (NSCLC) [[2], [3], [4]]. However, due to factors such as insufficient immune stimulatory signals and the immunosuppressive nature of the tumor microenvironment (TME), ICIs still face challenges, including a low immune response rate (10%-45%) [[5], [6], [7]]. In the hypoxic and necrotic cellular TME, a large amount of ATP is released, which is converted to adenosine by CD39 and CD73 on the surface of tumor cells [[8], [9], [10]]. Adenosine is a critical immunosuppressive metabolite and influences the activity of various immune cells, including dendritic cells (DCs), effector T cells, regulatory T cells (Tregs), and myeloid-derived suppressor cells (MDSCs), thereby severely inhibiting antitumor immune responses [11,12]. Inhibitors that target adenosine production or block its interaction with receptors have entered clinical trials [[13], [14], [15]]; however, these small molecular inhibitors encounter challenges such as targeted delivery.

Nanomedicines have been widely applied in the biomedical field, facilitating targeted drug delivery, reducing drug toxicity and side effects, synergize with immunotherapy, and achieving significant progress in cancer therapy [[16], [17], [18], [19], [20], [21], [22]]. Black phosphorus nanosheets (BPs), characterized by their large specific surface area, good photothermal properties, and biodegradability, have been utilized to develop a series of nanomedicines that integrate the modulation of immunosuppressive factors with photothermal therapy (PTT) to enhance immune responses [[23], [24], [25], [26]]. However, the application of these nanomedicines still faces significant challenges, such as loss during blood circulation and clearance by organs like the liver and kidneys, which prevent their efficient accumulation in tumors. Statistics indicate that the average tumor accumulation efficiency of nanomedicines in solid tumors is merely about 0.7%, with even lower efficiency in lung cancer [27]. Enhancing the accumulation of nanomedicines in lung cancer lesions is therefore a critical issue and a major challenge for improving therapeutic efficacy [[28], [29], [30]].

Cell-mediated drug delivery represents a novel strategy to enhance the tumor accumulation of nanomedicines. As the most abundant cell type in the bloodstream, with a circulation time of up to 120 days *in vivo*, red blood cells (RBC) have garnered increasing attention from researchers for their drug delivery capabilities [31,32]. RBC-based delivery systems, such as engineered RBC, RBC membranes, and RBC-derived vesicles, offer advantages including prolonged circulation time, high biocompatibility, and low immunogenicity [[33], [34], [35]]. By attaching nanomedicines to the surface of RBC, the targeted delivery efficiency of these nanotherapeutics can be improved through RBC-mediated transport *in vivo* [36,37]. Studies have shown that RBC-mediated delivery can alter the biodistribution and pharmacokinetic properties of nanomedicines, extending their *in vivo* circulation time by 2-5 fold and increasing their tumor accumulation efficiency by 5-10 fold [[38], [39], [40], [41], [42], [43]]. Although RBC-based nanomedicine platforms have seen progress, significant room for improvement remains in the strategies for conjugating nanocarriers to RBC and for subsequent drug release. For instance, traditional conjugation methods often rely on electrostatic interactions to adsorb nanomedicines onto the RBC surface. However, the relatively weak binding forces pose a risk of nanomedicine detachment during *in vivo* circulation.

Developing nanocarriers capable of stable binding with red blood cells and enabling responsive release upon specific biological or chemical signals in tissues could further expand the applications of RBC-based nanomedicines [44,45]. Aptamers, which bind to target molecules with high affinity and offer advantages such as low immunogenicity, minimal biotoxicity, ease of synthesis, and low cost, are widely used in the biomedical field and demonstrate promising application prospects [[46], [47], [48]]. During the screening and validation of aptamers, it is often observed that, in addition to their specific target molecules, aptamers may also bind to other molecules. For example, Sgc8, an aptamer targeting protein tyrosine kinase-7 (PTK7), has been found to potentially bind to other proteins such as ribonucleoproteins and keratins [49]; similarly, TD05, an aptamer targeting the immunoglobulin heavy Mu chain, may also bind to proteins including amino acid transporters and potassium voltage-gated channel proteins [50]. Another widely studied aptamer AS1411, was reported to be capable of binding to both nucleolin and actin with differential affinity [51,52].

Herein, we developed a novel biomimetic photothermal-immune nanomedicine delivery system utilizing RBC, BPs, the CD73 inhibitor adenosine 5'-(α, β-methylene) diphosphate (AMPCP), and the aptamer AS1411 (Apt). In this system, BPs are loaded with AMPCP and Apt. Apt mediates the binding of BPs to RBC, enabling long-term *in vivo* circulation and targeted accumulation in lung cancer through RBC hitchhiking mechanism. Leveraging the differential binding affinity of the aptamer, we achieve selective binding of BPs to RBC *versus* lung cancer cells, facilitating the release of the nanomedicine at the tumor site. By combining the photothermal effect of BPs with the adenosine inhibition of AMPCP, the anti-tumor immune response was boosted. The maturation of DCs was promoted by PTT and CD73 blockade, which also suppressed the Tregs cells and MDSCs, leading to increased activity of T lymphocytes. *Via* these combined effects, the lung cancer was converted from “cold tumor” to “hot tumor”, and its growth was significantly inhibited.

## 2 Result and discussion

### 2.1 Fabrication and characterization of AMPCP@Apt@BPs@RBC

**Fig. 1:**
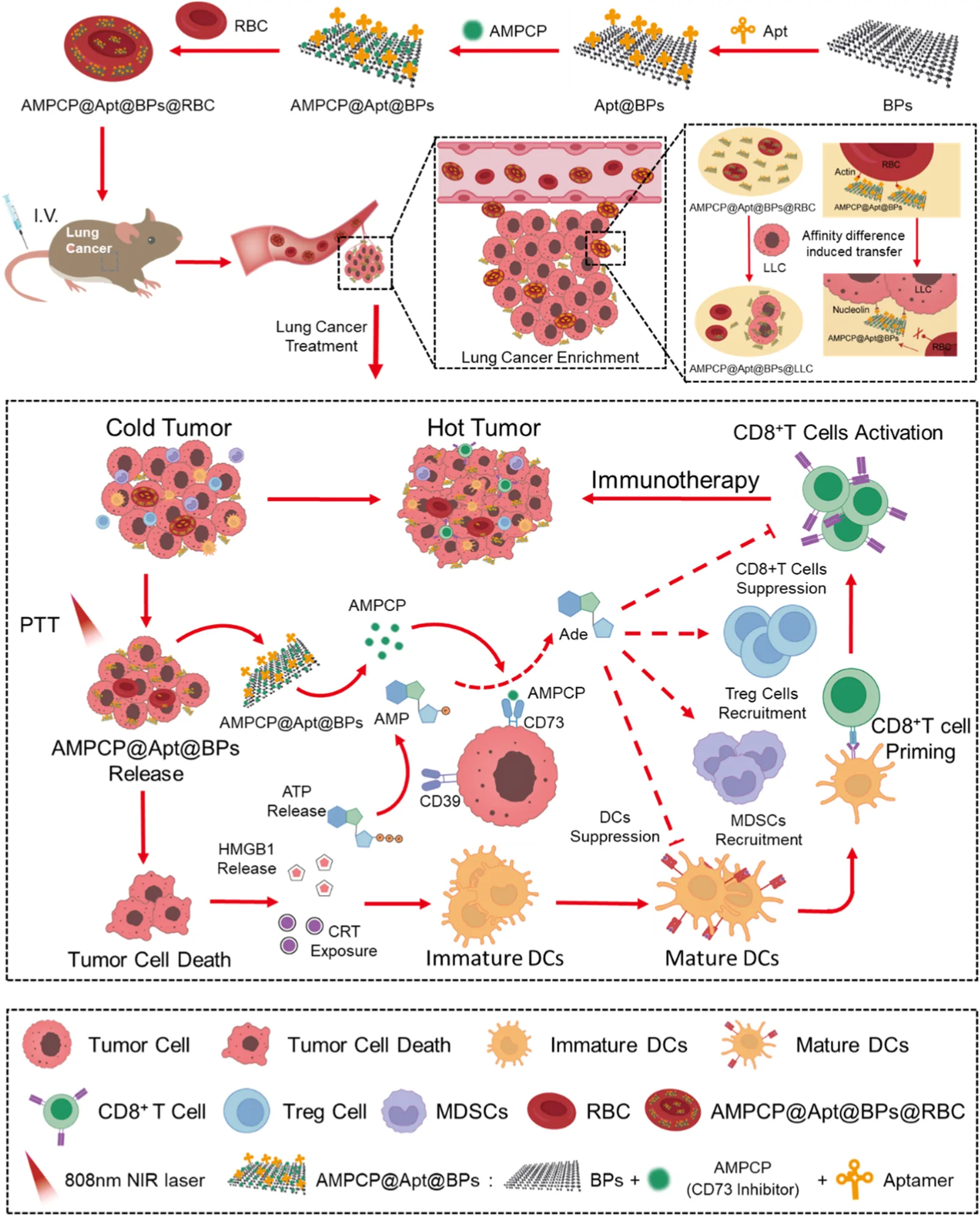
Schematic illustration of CD-73 blocking black phosphorus nanosheets hitchhiked on erythrocytes *via* affinity difference of Apt for photothermal-immunotherapy of lung cancer.

The preparation of AMPCP@Apt@BPs@RBC is illustrated in Fig. 1. Firstly, BPs were prepared according to our previously developed mechanical exfoliation method. Mn^2+^ ions were adsorbed onto the surface of BPs to modulate its surface charge. Subsequently, the AS1411 aptamer (Apt) and the CD73 inhibitor AMPCP were efficiently loaded onto the BPs' surface by mediation of Mn^2+^ to construct a photothermal nanomaterial, designated as AMPCP@Apt@BPs. This nanomaterial was then co-incubated with RBC. Through Apt-mediated binding of BPs to the RBC, the final complex AMPCP@Apt@BPs@RBC was formed.

**Fig. 2:**
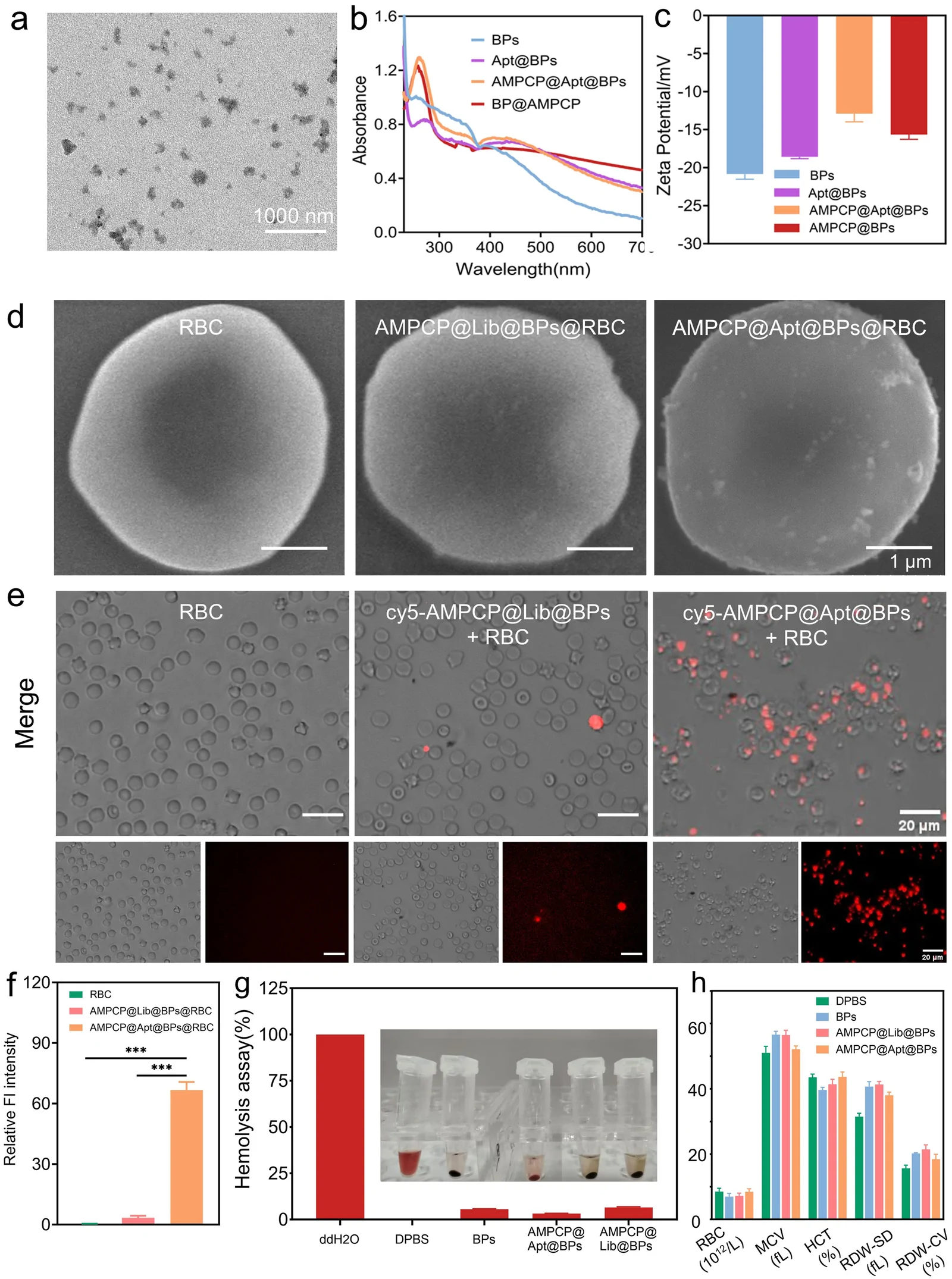
Fabrication and characterization of AMPCP@Apt@BPs@RBC. (a) TEM image of AMPCP@Apt@BPs. (b) UV-Vis-NIR absorption spectra, (c) Zeta potential measurements of BPs, Apt@BPs, and AMPCP@Apt@BPs. (d) SEM images of RBC, AMPCP@Lib@BPs@RBC, and AMPCP@Apt@BPs@RBC. Scale bar: 1 μm. (e) Fluorescence images, (f) Quantitative analysis of fluorescence signals of RBC after co-incubation with cy5-AMPCP@Lib@BPs and cy5-AMPCP@Apt@BPs, respectively. Red: cy5-labled Apt@BPs. Scale bar: 20 μm. (g) Hemolysis assay of the nanomaterials. (h) Routine blood test results. Data are presented as mean ± SD (n = 3). Statistical significance by one-way ANOVA: *P < 0.05, **P < 0.01, ***P < 0.001. (For interpretation of the references to colour in this figure legend, the reader is referred to the Web version of this article.)

The atomic force microscopy (AFM) image in Fig. S1 showed the topographic morphology of BPs, and the cross-sectional analysis determined that the thickness was 3.3 and 4.0 nm. The transmission electron microscopy (TEM) revealed that AMPCP@Apt@BPs were well-dispersed and uniform, with an average size of approximately 150 nm (Fig. 2a and Fig. S2). UV-Vis-NIR spectrophotometer was used to measure the absorption spectrum of AMPCP@Apt@BPs, together with its subcomponent materials AMPCP@BPs and Apt@BPs. The spectrum of AMPCP@BPs or Apt@BPs showed the characteristic absorption band of BPs (500-800 nm), along with distinct peaks corresponding to AMPCP or Apt (260 nm), indicating the successful loading of AMPCP or Apt on the BPs surface. For the spectrum of AMPCP@Apt@BPs, the absorbance at 260 nm was higher than that of AMPCP@BPs or Apt@BPs (Fig. 2b). Quantification of residual amounts in the supernatant indicated a loading efficiency of 96.48% for AMPCP and approximately 88.37% for Apt (Fig. S3). Changes in Zeta potential further validated the successful conjugation (Fig. 2c). The surface charge of BPs was negative, and changed slightly following attachment of Apt which has abundant phosphate groups. Upon subsequent coupling with AMPCP, the potential became less negative due to the surface occupation by the neutral AMPCP. In addition, the long-term storage stability of AMPCP@Apt@BPs was evaluated. As shown in Fig. S4, the zeta potential and diameter of the nanoparticles remained essentially unchanged after 7 days of storage at both 4 °C and −20 °C, and negligible drug leakage was observed under these conditions. These results indicated that AMPCP@Apt@BPs possess favorable long-term storage stability.

To further characterize the structure of AMPCP@Apt@BPs@RBC, the nanomaterial attached to the RBC surface after different treatments was observed using scanning electron microscopy (SEM). SEM imaging showed negligible nanoparticle attachment on untreated RBC, while a significant amount of nanomaterial was observed on AMPCP@Apt@BPs@RBC cocultured RBC. In contrast, only a small amount of nanomaterial was attached to RBC incubated with AMPCP@Lib@BPs@RBC, where BPs were modified with a random library aptamer (Lib) (Fig. 2d). This indicates that Apt mediates the binding of BPs to RBC, a process potentially related to the aptamer's ability to bind actin present on the RBC surface. Literature reports suggest that, in addition to targeting nucleolin, the Apt aptamer can also bind to actin [52]. Concurrently, cy5-labled AMPCP@Apt@BPs and AMPCP@Lib@BPs were prepared and co-incubated with RBC, and the presence of red fluorescent signal on RBC was assessed using microscope. As shown in Fig. 2e and f, no significant red fluorescence was observed in the RBC group and only a few RBC showed red fluorescence which treated with cy5-AMPCP@Lib@BPs. On the contrary, the majority of RBC cocultured with the AMPCP@Apt@BPs exhibited strong red fluorescent signal. These results further confirm that the Apt aptamer could mediate the binding of BPs to RBC.

Furthermore, the blood biocompatibility of the nanomaterial and the potential impact of nanomaterial attachment on RBC structure and function were investigated through hemolysis assays and routine blood tests. Hemolysis assay results showed no significant hemolysis during the co-incubation of the nanomaterial with RBC, indicating good blood biocompatibility (Fig. 2g). Routine blood test results demonstrated that nanomaterial attachment had a minimal effect on RBC structure, with all measured parameters in the nanomaterial-attached groups being close to those of the DPBS control group (Fig. 2h). These findings collectively suggest that the attachment of this nanomaterial does not significantly affect RBC structure.

### 2.2 Affinity difference induced transfer of BPs from erythrocytes to lung cancer cells

**Fig. 3:**
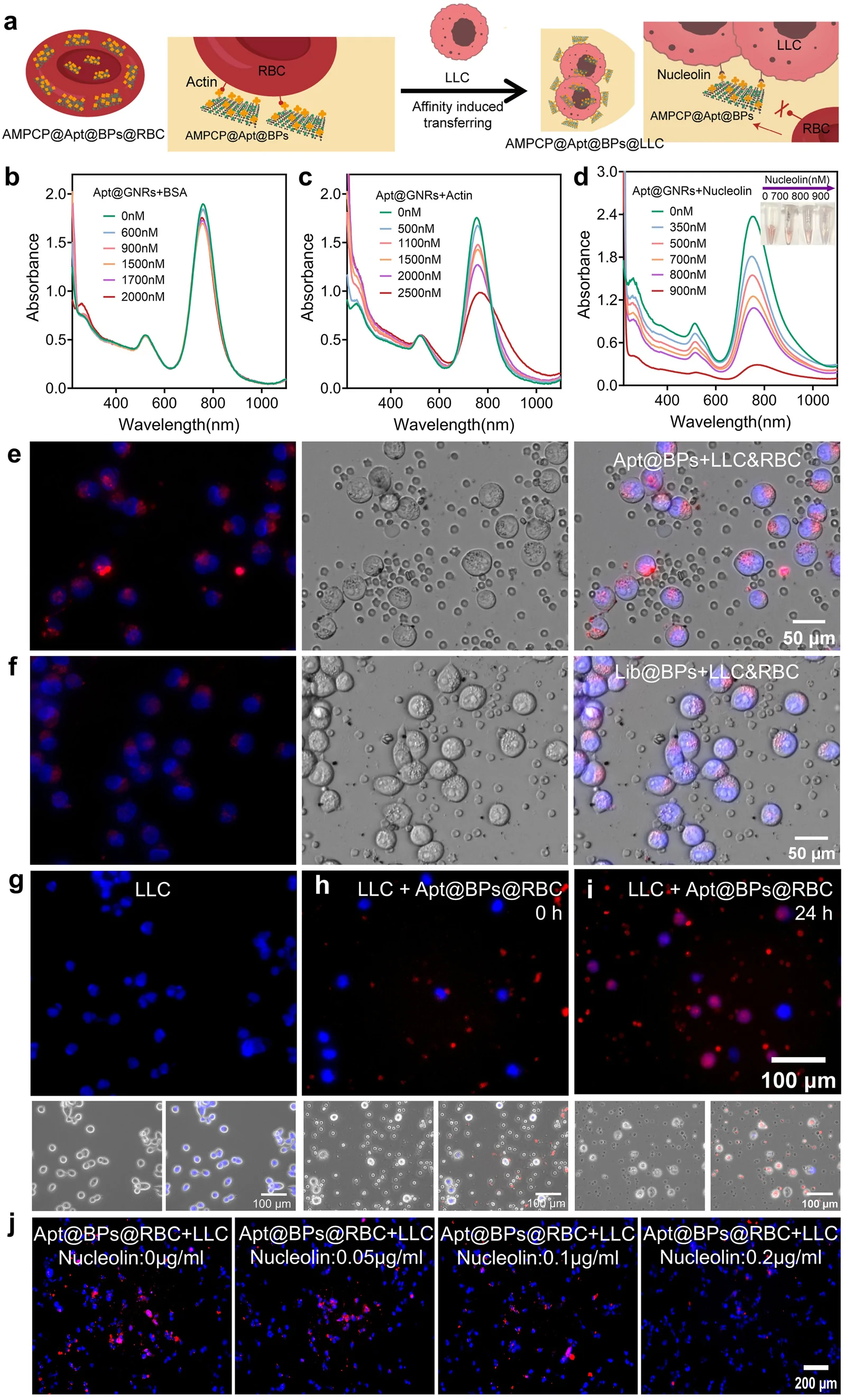
Affinity difference-induced transfer of BPs from erythrocytes to lung cancer cells. (a) Schematic illustration of *in vitro* transfer. UV-Vis-NIR absorption spectra of Apt@GNRs after incubation with various concentrations of (b) BSA, (c) Actin, and (d) Nucleolin. (e) Fluorescence image of LLC cells and RBC after co-incubation with cy5-labled Apt@BPs for 3 h. Blue: Hoechst; Red: cy5. (f) Fluorescence image of LLC cells and RBC after co-incubation with cy5-labled Lib@BPs for 3 h. Blue: Hoechst; Red: cy5. Fluorescence image of (g) LLC cells. (h) LLC cells co-incubated with cy5-labled Apt@BPs@RBC at 0 h, (i) LLC cells co-incubated with Apt@BPs@RBC after 24 h. (j) LLC cells co-incubated with Apt@BPs@RBC and different concentration of nucleolin. Blue: Hoechst; Red: cy5. (For interpretation of the references to colour in this figure legend, the reader is referred to the Web version of this article.)

Apt aptamer was reported to be able to bind both nucleolin and actin, exhibiting a higher affinity for nucleolin than actin [52]. Given the high expression of nucleolin on the surface of lung cancer cells, we hypothesized that this differential affinity could be exploited to achieve selective binding of BPs to RBC *versus* lung cancer cells, thereby facilitating the transfer of black phosphorus nanosheets from RBC to the tumor cell surface (Fig. 3a).

To validate the differential binding of Apt to nucleolin and actin, we synthesized gold nanorods (GNRs) and conjugated the 5′-thiol-modified Apt aptamer onto the GNRs surface *via* Au-S coordination to prepare Apt@GNRs (Fig. S5). GNRs have a unique longitudinal surface plasmon resonance (LSPR) band in the near-infrared (NIR) region, which can be changed by slight environmental variations around the GNRs, making GNRs as efficient biosensors for detection of specific targets and molecular interaction. GNRs and Apt@GNRs were incubated with varying concentrations of BSA, actin, and nucleolin, respectively. The binding capability of the aptamer to different proteins was assessed by monitoring changes in the UV-Vis-NIR absorption spectra.

As shown in Fig. S6, when unmodified GNRs were incubated with BSA, actin, or nucleolin, no significant changes in the LSPR peak were observed, indicating no substantial specific binding of GNRs to these proteins. For Apt@GNRs, the LSPR peak did not show a concentration-dependent decrease upon incubation with the control protein BSA, suggesting the absence of non-specific binding to general proteins (Fig. 3b). Fig. 3c demonstrates that with increasing concentrations of actin (0-2500 nM), the LSPR peak of Apt@GNRs gradually decreased, indicating that Apt@GNRs possess affinity for actin, consistent with literature reports of aptamer binding to actin [52]. Notably, incubation with much lower concentrations of nucleolin (0-900 nM) resulted in a more pronounced decrease in the LSPR peak of Apt@GNRs, demonstrating higher binding affinity of Apt@GNRs for nucleolin. These results confirm the differential affinity of the Apt aptamer for actin and nucleolin. Additionally, the binding affinity of coagulation factor, another protein recognized by the AS1411 aptamer, to Apt@GNRs was evaluated. Compared with nucleolin, coagulation factor displayed a considerably weaker affinity for Apt@GNRs, which was comparable to that of actin (Fig. S7).

Subsequently, cy5-labled Apt@BPs were co-incubated with a mixture of LLC cells and RBC. Observation *via* fluorescence microscopy revealed red fluorescence on the surface of LLC cells, while no red fluorescence was detected on RBC (Fig. 3e). This result indicated a higher affinity of Apt@BPs for LLC cells compared to RBC, likely attributable to the stronger binding of the aptamer to nucleolin on the LLC cell surface *versus* actin on the RBC surface. In contrast, the control nanomaterial Lib@BPs modified with a random library aptamer (Lib) showed very weak binding to both LLC cells and RBC (Fig. 3f and Fig. S8), ruling out non-specific binding effects of BPs to cells. These results demonstrate that leveraging the differential affinity of the aptamer enables selective binding of BPs to RBC and LLC cells.

To further verify whether this differential affinity could induce the transfer of BPs from RBC to LLC cells, cy5-labled Apt@BPs@RBC was prepared and was then co-incubated with LLC cells. Fluorescence intensities on different cell surfaces were monitored over time. Results showed that LLC cells alone exhibited no red fluorescence (Fig. 3g). Immediately upon co-incubation of LLC cells with Apt@BPs@RBC, red fluorescence was observed exclusively on RBC, with no signal on LLC cells (Fig. 3h). However, after 24 h of co-incubation, the red fluorescence on RBC diminished, while a strong red fluorescent signal appeared on the surface of a subset of LLC cells (Fig. 3i and Fig. S9), indicating the transfer of Apt@BPs from RBC to LLC cells. Additionally, this transfer was detected to be time-dependent. As shown in Fig. S10, the red fluorescence on LLC cells increased gradually from 4 h to 12 h. In order to further validate the affinity difference-driven transfer, different concentration of nucleolin were added into the incubation system. The results showed that the red fluorescence on LLC cells decreased with the adding of nucloelin (Fig. 3j), indicating less transfer of materials from RBC to LLC. This might be attributed to the competitive blockade of the interaction between the Apt and the nucleolin on LLC by the added nucleolin. Collectively, these findings demonstrated that the differential affinity of the aptamer can mediate the transfer of BPs from RBC to LLC cells.

### 2.3 *In vitro* photothermal ablation and immune induction of AMPCP@Apt@BPs@RBC

**Fig. 4:**
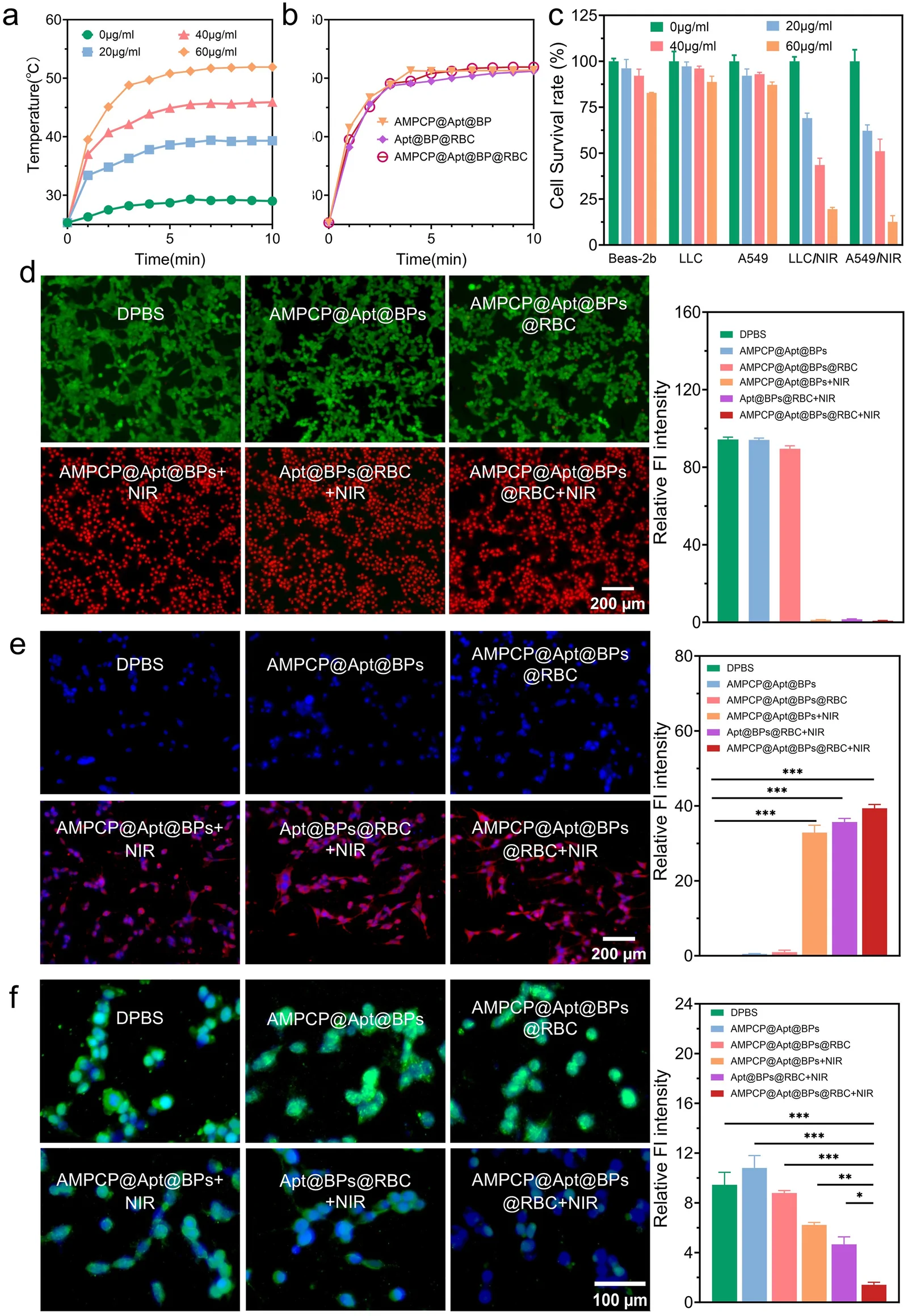
*In vitro* photothermal ablation and immune induction by AMPCP@Apt@BPs@RBC. (a) Temperature elevation profiles of AMPCP@Apt@BPs@RBC with different BPs concentrations upon laser irradiation for 10 min. (b) Temperature elevation profiles of various formulations upon laser irradiation for 10 min. (c) CCK-8 assay results for Beas-2b, LLC, and A549 cells co-cultured with AMPCP@Apt@BPs@RBC with different BPs concentrations and subjected to different treatments. (d) *In vitro* photothermal ablation of LLC cells under different treatment by Calcein-AM/PI staining. Green: Calcein-AM; red: PI; n = 3. (e) CRT expression on the LLC cell membrane and corresponding quantitative fluorescence analysis. Blue: Hoechst; red:AF549. (f) HMGB1 release from the nucleus of LLC cells and corresponding quantitative fluorescence analysis. Blue: Hoechst; Green:AF488. Data are presented as mean ± SD (n = 3). Statistical significance by one-way ANOVA: *P < 0.05, **P < 0.01, ***P < 0.001. (For interpretation of the references to colour in this figure legend, the reader is referred to the Web version of this article.)

The photothermal capacity of AMPCP@Apt@BPs@RBC was evaluated. Upon irradiation with an 808 nm near-infrared laser at a power density of 1 W cm^−2^ for 10 min, all of the temperature of various formulations containing BPs at a concentration of 60 μg/mL increased to approximately 50 °C, including AMPCP@Apt@BPs, Apt@BPs@RBC, and AMPCP@Apt@BPs@RBC. This confirms that conjugation with RBC does not compromise the photothermal efficiency of the BPs (Fig. 4a). Subsequently, the temperature elevation of AMPCP@Apt@BPs@RBC at different BPs concentrations was measured under the same laser irradiation conditions. After 10 min, the solution temperature reached approximately 39.3 °C at a BPs concentration of 20 μg/mL, 45.9 °C at 40 μg/mL, and 51.9 °C at 60 μg/mL (Fig. 4b). These results demonstrate that AMPCP@Apt@BPs@RBC possesses good photothermal properties *in vitro*.

Next, the *in vitro* cytotoxic effect of AMPCP@Apt@BPs@RBC on cells was investigated. Initially, normal Beas-2b cells were co-incubated with varying concentrations of AMPCP@Apt@BPs@RBC. No significant effect on Beas-2b cell viability was observed (Fig. S11), indicating that the material itself has negligible inherent toxicity. Subsequently, LLC cells were then co-incubated with different materials, concentrations of AMPCP@Apt@BPs@RBC and subsequently exposed to 808 nm laser irradiation (1 W cm^−2^) for 10 min. CCK-8 assay results showed that following photothermal treatment at a BPs concentration of 60 μg/mL, the cell viability of LLC was only 18.88%, and that of A549 cells was only 12.61% (Fig. 4c). Calcein-AM/PI staining results were consistent with the CCK-8 assay, demonstrating a concentration-dependent increase in photothermal killing of LLC and A549 cells, culminating in nearly complete cell death at the highest concentration (Fig. S12). The inhibitory effect of AMPCP@Apt@BPs@RBC to LLC cells was further investigated with comparison of various materials and treatment. As shown in Fig. 4d, treatment of materials *plus* NIR irradiation caused obvious death of LLC cells, comparing to those without PTT treatment. Additionally, the photothermal efficacy of AMPCP@Apt@BPs@RBC in killing B16F10 melanoma cells was also studied, which showed similar ablation effect with LLC cells (Fig. S13). These findings indicate that AMPCP@Apt@BPs@RBC exerts a potent killing effect on tumor cancer cells *in vitro*.

Furthermore, the *in vitro* immunogenic induction capacity of AMPCP@Apt@BPs@RBC was explored. PTT is known to immunogenic cell death (ICD), which is typically characterized by hallmarks such as the translocation of calreticulin (CRT) to the cell surface and the release of high mobility group box 1 protein (HMGB1). Therefore, the level of CRT and HMGB1 was assessed to evaluate the immunogenic induction capability of AMPCP@Apt@BPs@RBC *in vitro*. Fluorescence microscopy revealed that following near-infrared laser irradiation of LLC cells treated with the nanomedicine, CRT expression on the cell membrane was increased (Fig. 4e), while HMGB1 fluorescence intensity within the nucleus was significantly diminished (Fig. 4f). The Enzyme-Linked Immunosorbent Assay (ELISA) measurement of HMGB1 was consistent with the fluorescence test (Fig. S14). Furthermore, similar upregulation of CRT (Fig. S15) and downregulation of HMGB1 (Fig. S16) were observed in A549 cells, confirming that AMPCP@Apt@BPs@RBC possesses a robust immunogenic induction capacity *in vitro*.

Except for the induction of CRT expression and HMGB1 release, the activation of dendritic cells by AMPCP@Apt@BPs@RBC was further investigated. Bone marrow cells were extracted from C57 mice and induced differentiation to acquire the immature dendritic cells (iDCs). Then the iDCs were cultured in the lower chamber of Transwell plates, with LLC cells and AMPCP@Apt@BPs@RBC in the upper channel. The upper channel was irradiated with 808 nm laser upon power of 1.0 W cm^−2^ for 5 min. The DCs in the lower chamber was collected after irradiation and stained for flow cytometric analysis. As shown in Fig. S17, the LLC cells treated with AMPCP@Apt@BPs and AMPCP@Apt@BPs@RBC can both slightly increase the maturation of iDCs, which was further raised by the photothermal irradiation. Treatment of AMPCP@Apt@BPs@RBC + NIR could facilitate the iDCs maturation to 58.1%, which was significantly higher than the untread control (5.3%). These results demonstrated that the photothermal effect of AMPCP@Apt@BPs@RBC can promote the activation of DCs.

### 2.4 *In vivo* anti-tumor effect of AMPCP@Apt@BPs@RBC

**Fig. 5:**
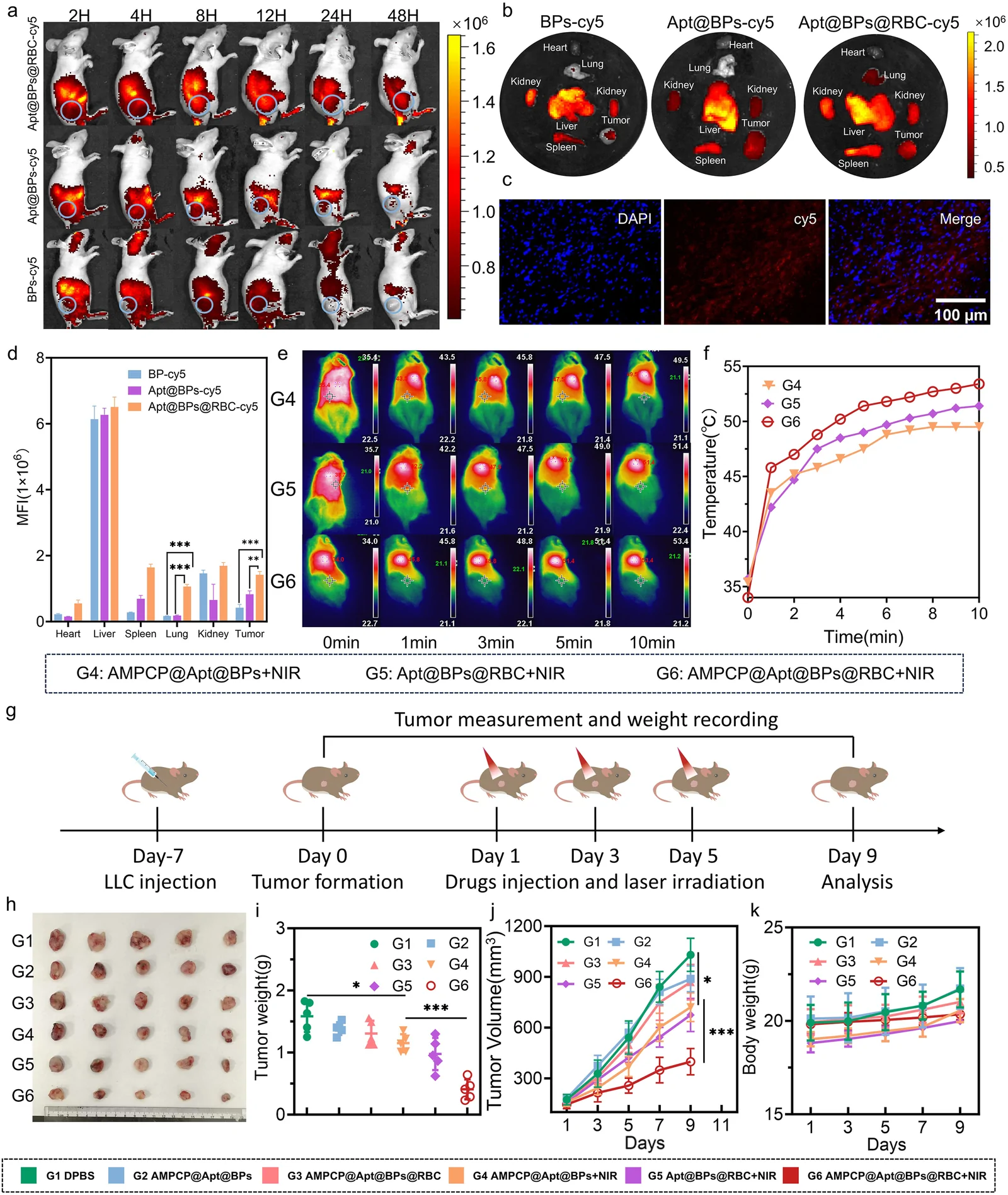
*In vivo* anti-tumor effect of AMPCP@Apt@BPs@RBC. (a) *In vivo* imaging of tumor-bearing nude mice at different time points after intravenous injection of nanomaterials. Red: cy5. (b) *Ex vivo* biodistribution of nanomaterials in tumors and major organs 8 h post-injection. Red: cy5. (c) Fluorescence image of tumor cryosection from a tumor-bearing nude mouse 96 h post-injection. (d) Quantitative fluorescence analysis of nanomaterial distribution in tumors and major organs. (e) Temperature changes and infrared thermal images, (f) Quantification of the tumor region in tumor-bearing C57 mice from different groups during NIR irradiation. (g) Schematic illustration of the animal study timeline and treatment regimen. (h) Photographs of excised tumors at the end of the treatment period. (i) Tumor weights measured on the ninth day. Changes in (j) tumor volume, (k) Body weight throughout the treatment period. (G1) DPBS, (G2) AMPCP@Apt@BPs, (G3) AMPCP@Apt@BPs@RBC, (G4) AMPCP@Apt@BPs + NIR, (G5) Apt@BPs@RBC + NIR, and (G6) AMPCP@Apt@BPs@RBC + NIR. Data are presented as mean ± SD (n = 5). Statistical significance by one-way ANOVA: *p < 0.05, **p < 0.01, ***p < 0.001. (For interpretation of the references to colour in this figure legend, the reader is referred to the Web version of this article.)

Next, the *in vivo* targeting capability and circulation time of the system were evaluated. Freshly prepared cy5-labeled formulations were intravenously injected into tumor-bearing nude mice, and *in vivo* fluorescence imaging was performed at designated time points. As shown in Fig. 5a, compared to the BPs group and the RBC-free Apt@BPs group, the Apt@BPs@RBC group exhibited enhanced targeted accumulation at the tumor site and a significantly prolonged *in vivo* circulation time. At various time points (2, 4, 8, 12, 25, and 48 h post-injection), the fluorescence signal in the tumor tissue of the Apt@BPs@RBC group was consistently higher than that in the BPs and Apt@BPs groups. *Ex vivo* imaging of organs at 8 h post-injection and subsequent quantitative analysis revealed a pronounced accumulation of Apt@BPs@RBC in the tumor (Fig. 5b and d). Apart from the tumor, an elevated fluorescence signal was observed in the lung of the Apt@BPs@RBC group compared to BPs and Apt@BPs groups, likely reflecting the biodistribution characteristics of the RBC biomimetic delivery platform [43]. Similar signal level was obtained in the liver, perhaps due to that the liver serves as the predominant organ for nanomaterial sequestration and loading the nanoparticles onto erythrocytes did not appreciably reduce their hepatic deposition. Notably, strong fluorescence from Apt@BPs@RBC was still detectable in the tumor tissue at 48 h, 72 h and 96 h (Fig. 5a and Fig. S18), whereas most of the fluorescence signals from BPs and Apt@BPs diminished at 48 h, reflecting the long-circulation effect of RBC hitchhiking. Cryosectioning of tumor tissue collected 96 h post-injection confirmed the persistent accumulation of Apt@BPs@RBC (Fig. 5c and Fig. S19), further validating the tumor-targeting efficacy and prolonged *in vivo* circulation conferred by this system. Similar bio-distribution of materials in C57 mice was observed (Fig. S20), showing long-term enrichment of Apt@BPs@RBC in tumor. These results demonstrated that the RBC hitchhiking strategy mediated by the aptamer altered the biodistribution, extended the circulation time, and enhanced the accumulation of the nanomaterial in the tumor region.

Subsequently, the *in vivo* photothermal effect of the system was assessed. The formulations were intravenously injected into tumor-bearing C57 mice. 4 h post-injection, the tumor area was irradiated with an 808 nm near-infrared laser at a power density of 0.65 W cm^−2^ for 10 min, and temperature changes were monitored using a photothermal imaging camera. The results showed that the tumor temperature in the AMPCP@Apt@BPs@RBC + NIR group reached 53.4 °C upon laser irradiation, which was higher than that observed in the AMPCP@Apt@BPs + NIR group at 49.5 °C and the Apt@BPs@RBC + NIR group at 51.4 °C (Fig. 5e and f). This enhanced photothermal effect is likely attributable to the improved accumulation of BPs in the tumor region facilitated by the RBC hitchhiking strategy.

Finally, the *in vivo* antitumor efficacy of AMPCP@Apt@BPs@RBC was evaluated. C57 mice were inoculated subcutaneously in the right armpit with LLC cells. When the tumor volume reached approximately 200 mm^3^, the tumor-bearing mice were randomly divided into six different treatment groups (n = 5 per group): (G1) DPBS control, (G2) AMPCP@Apt@BPs, (G3) AMPCP@Apt@BPs@RBC, (G4) AMPCP@Apt@BPs *plus* NIR irradiation, (G5) Apt@BPs@RBC *plus* NIR irradiation, and (G6) AMPCP@Apt@BPs@RBC *plus* NIR irradiation. Treatments were administered every other day for a total of three doses. Body weight and tumor volume were recorded throughout the treatment period. Following the final treatment, mice were rested for one week before being euthanized. Tumors were promptly excised, weighed, and photographed.

As shown in Fig. 5g–j, mice in the AMPCP@Apt@BPs@RBC + NIR group (G6) exhibited the smallest tumor weight and volume, significantly lower than those in the untreated control group (G1), the AMPCP@Apt@BPs alone group (G2), the AMPCP@Apt@BPs@RBC alone group (G3), the AMPCP@Apt@BPs + NIR group (G4), and the Apt@BPs@RBC + NIR group (G5). Comparing with the DPBS group which showed the largest tumor volume (1030.53 mm^3^), the AMPCP@Apt@BPs@RBC + NIR group exhibited a 2.58-fold of tumor size reduction (398.85 mm^3^). These results demonstrate that the RBC-mediated targeted accumulation, combined with the synergistic effect of AMPCP-mediated adenosine inhibition and the photothermal effect of BPs, effectively suppressed tumor growth. Throughout the treatment course, mouse body weights were monitored. The results showed a steady increase of body weight with parallel growth trajectories across the groups (Fig. 5k).

### 2.5 Immunoregulatory effect of AMPCP@Apt@BPs@RBC

To further investigate the immunomodulatory effects of AMPCP@Apt@BPs@RBC, the ATP level in tumor site was measured. As shown in Fig. S21, in NIR irradiation groups (G4, G5, and G6), tumor ATP levels were increased by 1.87-fold, 1.53-fold, and 2.19-fold, respectively, compared with the DPBS group (G1), whereas no significant changes were observed in the groups that did not receive laser irradiation (G2 and G3). This was consistent with the reported phenomenon that the PTT would elicit the production of ATP in tumor.

The adenosine level of the tumour tissue was next examined. As displayed in Fig. S22, photothermal treatment with Apt@BPs@RBC (G5) increased the level of adenosine in tumor to a certain extent. However, the involvement of AMPCP in the photothermal treatment could inhibit the rise of adenosine, as illustrated in the AMPCP@Apt@BPs + NIR (G4) and AMPCP@Apt@BPs@RBC + NIR (G6) groups. Moreover, the inhibitory effect of AMPCP@Apt@BPs@RBC + NIR was even higher than that of AMPCP@Apt@BPs + NIR, which may be speculated to the enhanced accumulation of the nanomedicine in tumor induced by erythrocyte hitchhiking.

**Fig. 6:**
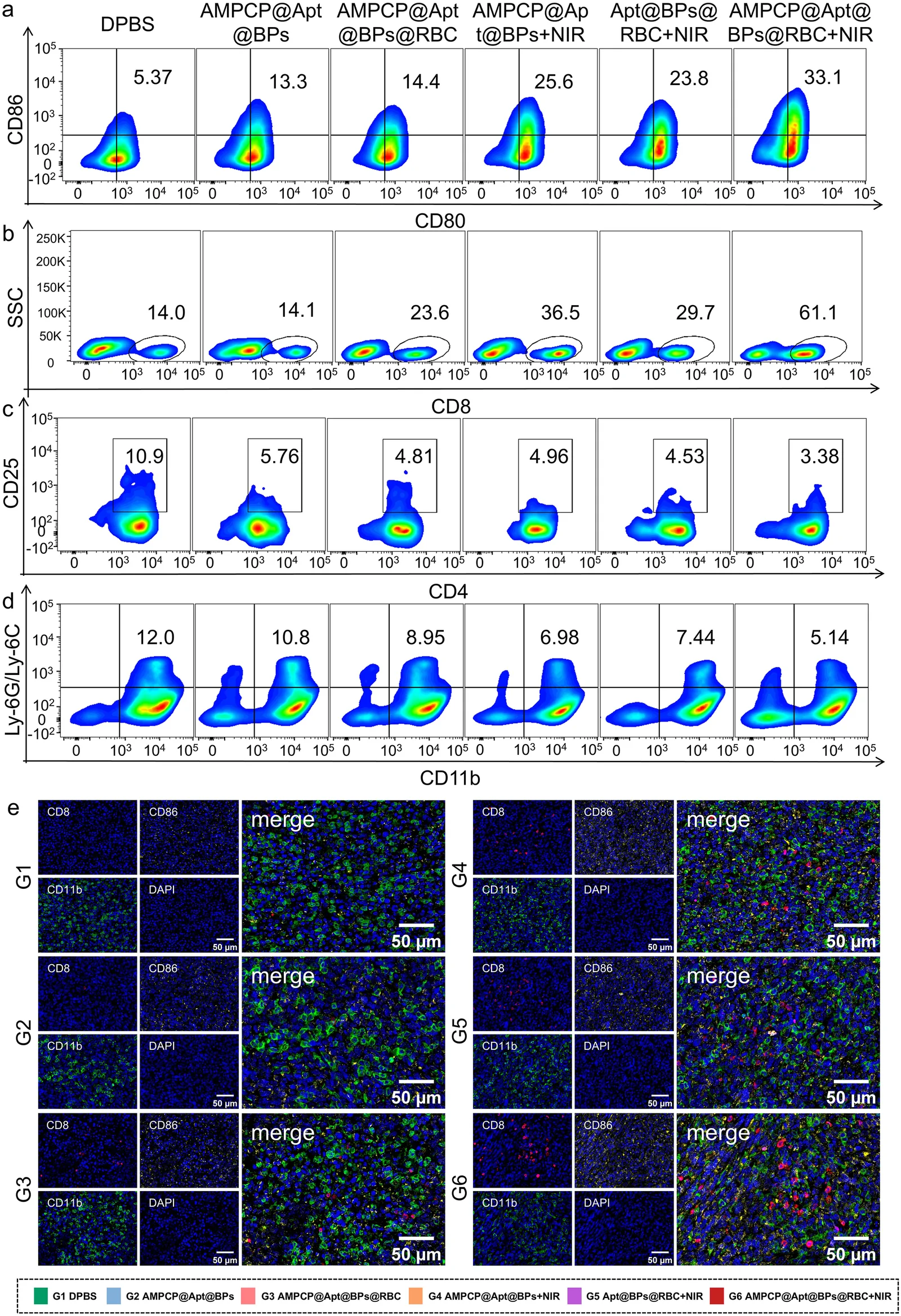
Immunoregulatory effect of AMPCP@Apt@BPs@RBC. Flow cytometry analysis of (a) CD80^+^ CD86^+^ DCs, (b) CD8^+^ T cells, (c) Tregs, (d) MDSCs. (e) Immunofluorescence staining of T cells, DCs, Tregs, and MDSCs in tumor tissue sections. (G1) DPBS, (G2) AMPCP@Apt@BPs, (G3) AMPCP@Apt@BPs@RBC, (G4) AMPCP@Apt@BPs + NIR, (G5) Apt@BPs@RBC + NIR, and (G6) AMPCP@Apt@BPs@RBC + NIR.

Subsequently, flow cytometry analysis and immunostaining of immune related cells were performed. Dendritic cells play a crucial role in antigen presentation and immune response, functioning in both antigen presentation and immune regulation. The promotion of DCs maturation by AMPCP@Apt@BPs@RBC was evaluated by assessing the DCs population in tumor tissues *via* flow cytometry (Fig. S23). As shown in Fig. 6a, comparing to the proportion of CD80^+^ CD86^+^ DCs in the DPBS group (5.37%), the DC maturation in AMPCP@Apt@BPs group (G2), and AMPCP@Apt@BPs@RBC group (G3) was increased to 13.3% and 14.4% respectively, revealing the effect of AMPCP in promoting DC maturation. The AMPCP@Apt@BPs + NIR group (G4) showed an increased proportion of CD80^+^ CD86^+^ DCs (25.6%), indicating the combined effect of AMPCP and the photothermal effect of BPs. Notably, the Apt@BPs@RBC + NIR group (G5), although lacking AMPCP, also exhibited an elevated proportion of CD80^+^ CD86^+^ DCs (23.8%), attributable to enhanced targeting mediated by RBC. The AMPCP@Apt@BPs@RBC + NIR group (G6), which integrated RBC-mediated targeting, AMPCP, and the photothermal effect of BPs, demonstrated the most substantial enhancement in CD80^+^ CD86^+^ DCs (33.1%). Compared with the control group (DPBS), the proportion of dendritic cells (DCs) in the AMPCP@Apt@BPs@RBC + NIR group increased by 6.16-fold (Fig. S24).

DCs can promote the generation of cytotoxic T lymphocytes (CTLs). As CTLs, CD8^+^ T cells can directly kill cancer cells. Changes in CD8^+^ T cells within tumor tissues were assessed by flow cytometry to further evaluate the immunomodulatory effects of AMPCP@Apt@BPs@RBC (Fig. S25). As shown in Fig. 6b, compared to the control group, the G2 group showed no significant change in CD8^+^ T cells. However, mediated by RBC hitchhiking, the G3 group exhibited a moderate increase in the proportion of CD8^+^ T cells. The proportion of CD8^+^ T cells was further enhanced in the G4 group combining AMPCP and photothermal effects (2.62-fold) and the G5 group combining RBC hitchhiking and photothermal effects (2.12-fold). The most significant increase in the proportion of CD8^+^ T cells was observed in the G6 group, which integrated effects of AMPCP, PTT, and RBC hitchhiking (4.36-fold, Fig. S24b).

Regulatory T cells are among the major immunosuppressive cell populations in TME. The impact of the system on Tregs was assessed by measuring the levels of two key biomarkers (CD4 and CD25) in tumor tissues *via* flow cytometry (Fig. S25). As shown in Fig. 6c, the G2 group reduced the proportion of Tregs from 10.9% to 5.76%, indicating the effect of AMPCP on inhibiting Tregs. Following near-infrared laser irradiation, the intratumoral Tregs proportion was decreased to 4.96% in the G4 group, revealing the combined effect of AMPCP and PTT on suppressing Tregs. With RBC hitchhiking-mediated delivery, the proportion was further reduced to 4.81% in the G3 group. The most pronounced reduction was observed in the AMPCP@Apt@BPs@RBC + NIR treatment group, which combined AMPCP, PTT and RBC hitchhiking all together (G6, Fig. S24c).

Myeloid-derived suppressor cells represent another major immunosuppressive cell population within the TME. The effect of the system on MDSCs was evaluated by detecting the MDSCs marker CD11b using flow cytometry (Fig. S26). Fig. 6d shows that AMPCP@Apt@BPs reduced the proportion of MDSCs from 12.0% to 10.8%. With RBC hitchhiking-mediated delivery, the proportion was further reduced to 8.95% in the G3 group. Upon near-infrared laser irradiation, the MDSC proportions in the Apt@BPs@RBC + NIR, AMPCP@Apt@BPs + NIR, and AMPCP@Apt@BPs@RBC + NIR groups were reduced to 7.44%, 6.98%, and 5.14%, respectively (Fig. S24d).

The changes in various cell populations within the tumor tissue was further analyzed using immunofluorescence staining. As shown in Fig. 6e and Fig. S27, the AMPCP@Apt@BPs@RBC + NIR group exhibited the most pronounced enhancement of CD80^+^ CD86^+^ DCs and CD8^+^ T cells, along with the most marked reduction in CD4^+^ CD25^+^ Tregs and CD11b^+^ MDSCs. The trends observed in the other groups were consistent with the flow cytometry results, further validating the system's ability to modulate the immune microenvironment and promote anti-tumor immunity.

### 2.6 Abscopal anti-tumor effect of AMPCP@Apt@BPs@RBC

**Fig. 7:**
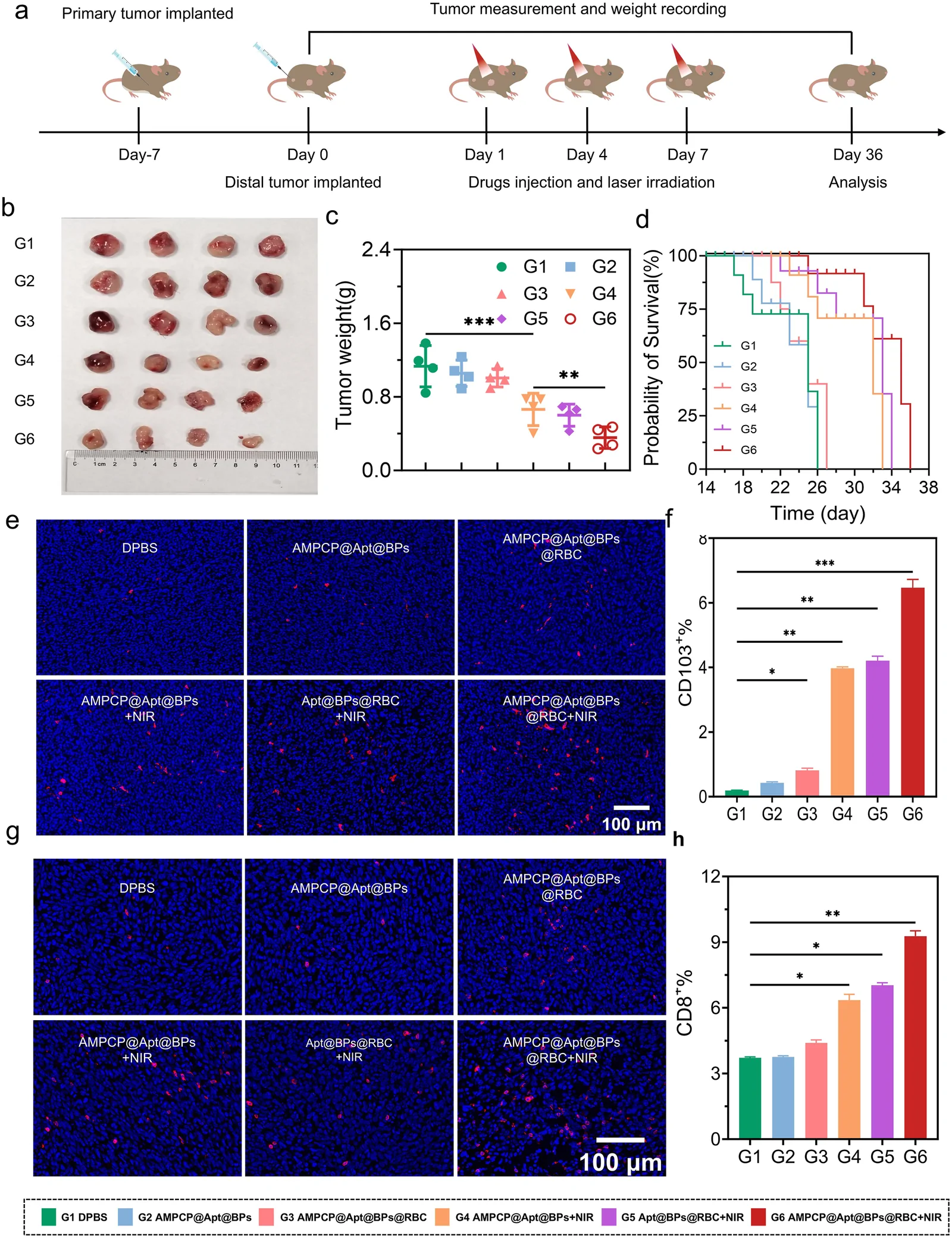
Abscopal anti-tumor effect of AMPCP@Apt@BPs@RBC. (a) Schematic diagram of the treatment protocol. (b) Pictures of distal tumors exfoliated from different groups of mice at the end of the treatment. (c) Tumor weights of the distal tumor after the treatment. (d) Mice survival curves in each treatment group. (e) Immunofluorescence staining, and (f) quantitative analysis of CD103^+^ memory T cells in the primary tumor tissue sections. (g) Immunofluorescence staining, and (h) quantitative analysis of CD8^+^ T cells in the distal tumor tissue sections. (G1) DPBS, (G2) AMPCP@Apt@BPs, (G3) AMPCP@Apt@BPs@RBC, (G4) AMPCP@Apt@BPs + NIR, (G5) Apt@BPs@RBC + NIR, and (G6) AMPCP@Apt@BPs@RBC + NIR. Data are presented as mean ± SD (n = 4). Statistical significance by one-way ANOVA: *p < 0.05, **p < 0.01, ***p < 0.001.

Based on the immune modulation ability of AMPCP@Apt@BPs@RBC, its abscopal effect on suppressing distal tumor by was further evaluated. As shown in Fig. 7a, a distal tumor was implanted on the other side of the primary tumor on mice. The primary tumor was irradiated by laser, and the volume of the distal tumor was monitored. The distal tumors of the mice in the AMPCP@Apt@BPs@RBC + NIR group exhibited the smallest tumor volume and weight comparing with all other groups, illustrating the potential distal tumor inhibition effect rendered by AMPCP@Apt@BPs@RBC + NIR (Fig. 7b and c). Moreover, this treatment also significantly prolonged mice survival compared with other groups (Fig. 7d).

Given that immune memory is critical for preventing tumor metastasis, the presence of CD103^+^ memory T cells in primary tumors was assessed *via* immunofluorescence staining. As shown in Fig. 7e and f, treatment with AMPCP@Apt@BPs@RBC + NIR elevated the level of CD103^+^ T cells within the primary tumor. Furthermore, the CD8^+^ T cell population in the distal tumor microenvironment was also analyzed. As presented in Fig. 7g and h, the abundance of CD8^+^ T cells in distal tumors was markedly increased following the same treatment. Such immune stimulating efficacy was stronger than other controlling groups. Collectively, these results indicated that an efficient systemic anti-tumor immune response was generated to inhibit the distal tumor.

### 2.7 Biosafety study of AMPCP@Apt@BPs@RBC

**Fig. 8:**
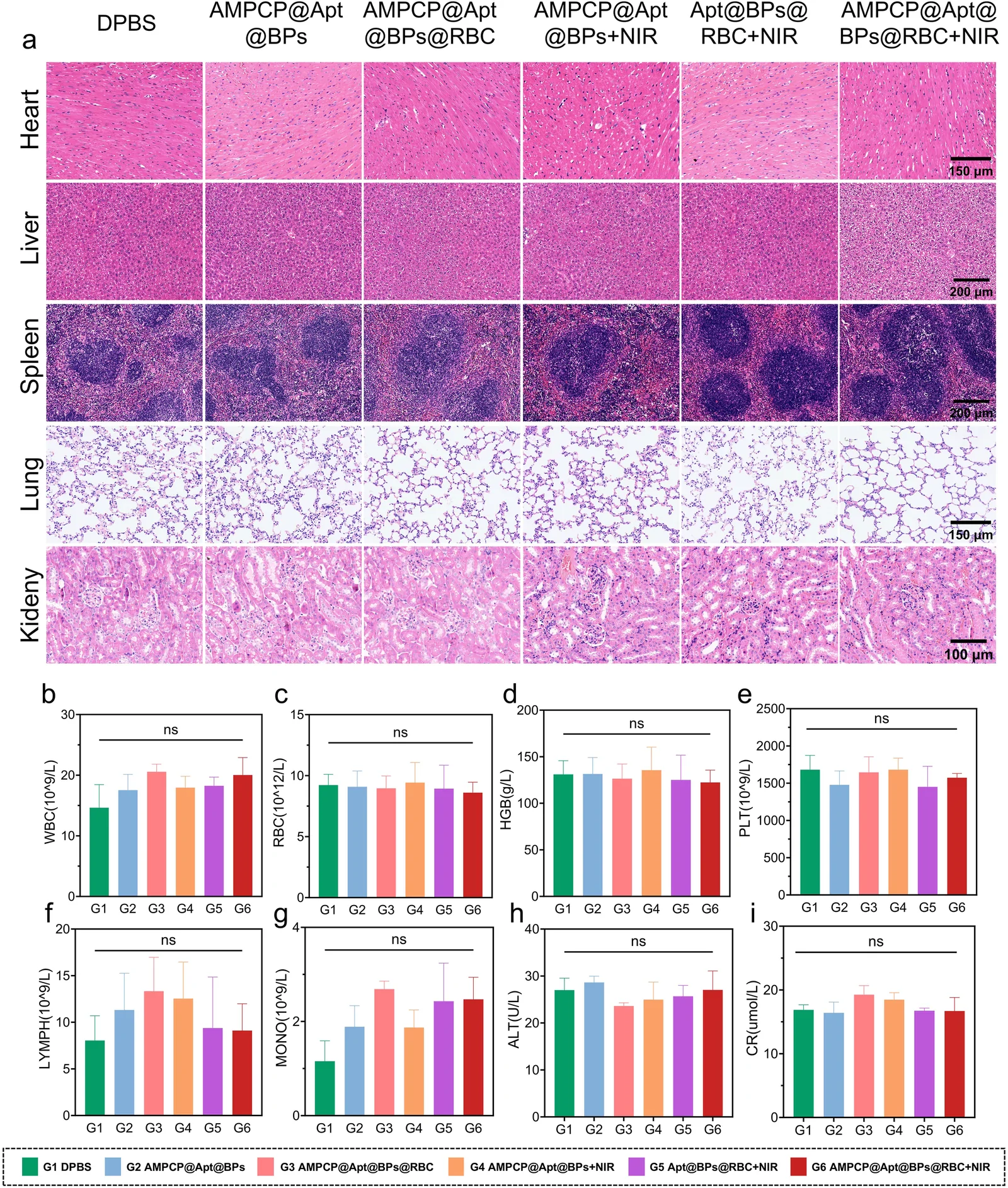
Biosafety study of AMPCP@Apt@BPs@RBC. (a) H&E staining of major organs (heart, liver, spleen, lungs, kidneys). (b) WBC count, (c) RBC count, (d) HGB level, (e) PLT count, (f) LYMPH count, (g) MONO count in whole blood. (h) ALT, (i) CR level in peripheral blood. (G1) DPBS, (G2) AMPCP@Apt@BPs, (G3) AMPCP@Apt@BPs@RBC, (G4) AMPCP@Apt@BPs + NIR, (G5) Apt@BPs@RBC + NIR, and (G6) AMPCP@Apt@BPs@RBC + NIR. Data are presented as mean ± SD (n = 5).Statistical significance by one-way ANOVA: *P < 0.05, **P < 0.01, ***P < 0.001.

Following the treatment period, the biosafety of this therapeutic strategy was further evaluated. As shown in Fig. 8a–H&E staining of sections from the heart, liver, spleen, lungs, and kidneys revealed no significant abnormalities, indicating that the major organs of the mice were not noticeably damaged. The captured image of spleen showed that the morphology and size of spleen were not affected by the treatment (Fig. S28). To evaluate the risk of photothermia to the surrounding tissues, the skin and muscle near the laser-irradiating site was collected for H&E staining. As shown in Fig. S29, no obvious damage of skin and muscle was observed, either after once or three times of laser irradiation. Furthermore, Changes in blood cell counts were measured using a hematology analyzer to further assess whether the mice experienced infection or inflammation during the treatment process (Fig. 8b–g). The observed differences in white blood cells (WBC), red blood cells (RBC), hemoglobin (HGB), platelets (PLT), absolute lymphocyte count (LYMPH), and absolute monocyte count (MONO) were not statistically significant, suggesting the absence of infection or inflammation throughout the treatment course. Finally, the levels of alanine aminotransferase (ALT), creatinine (CR), aspartate aminotransferase (AST), blood urea nitrogen‌ (BUN) and Uric Acid (UA) in peripheral blood were analyzed (Fig. 8h–i, and Fig. S30). The results showed that these parameters remained essentially normal within the safety range, indicating no significant impairment of liver and kidney function in the mice.

## 3 Conclusion

In summary, this study successfully constructed an erythrocytes-based photothermal-immune nanomedicine delivery vehicle, designated as AMPCP@Apt@BPs@RBC, by integrating red blood cells, AS1411 aptamer, black phosphorus nanosheets, and the CD73 inhibitor AMPCP. This system leverages RBC hitchhiking to prolong the blood circulation time of the nanomedicine and enhance its targeting to lung cancer. The binding of the black phosphorus-based nanomedicine to RBC was achieved through the affinity of the aptamer for actin on the RBC surface. Furthermore, exploiting the different affinity of the aptamer for nucleolin *versus* actin facilitated the transfer of the nanomedicine from RBC to lung cancer cells, enabling its release specifically at the tumor site to exert its therapeutic function. AMPCP@Apt@BPs@RBC demonstrated efficient accumulation in tumor tissues, generating a potent photothermal effect that not only effectively ablated tumor cells but also induced immunogenic cell death to activate antitumor immunity. BPs and AMPCP synergistically induce the maturation of dendritic cells, resulting in a 6.16-fold increase in their proportion. Furthermore, they promote a 4.36-fold expansion of CD8^+^ T cells while reducing the proportions of regulatory T cells and myeloid-derived suppressor cells by 3.22-fold and 2.33-fold, respectively, thereby reshaping the immunosuppressive microenvironment and enhancing antitumor immune responses. This combined therapeutic strategy of photothermal therapy and immunotherapy elicits potent immune stimulation and effectively suppresses lung cancer proliferation, with the mean tumor volume reduced by 2.58-fold. This aptamer-guided, CD73-blocing black phosphorus nanosheet hitchhiked on RBC delivery system, not only facilitates the targeted accumulation of nanomedicines but also elicits robust antitumor immune activation, providing an effective nanoplatform for suppression of lung cancer.

## CRediT authorship contribution statement

**Qiao Liu:** Investigation, Writing – original draft. **Niu Liu:** Investigation. **Zhiqi Lao:** Funding acquisition, Investigation, Writing – review & editing. **Jian Qin:** Funding acquisition, Writing – review & editing. **Yuting Zhong:** Data curation, Investigation. **Kaiwei Hou:** Data curation, Investigation. **Yuetao Zhao:** Conceptualization, Funding acquisition, Supervision, Writing – review & editing.

## Ethics approval and consent to participate

All SPF-grade mice used in the experiments were purchased from Hunan SJA Laboratory Animal Co., Ltd., and housed under standard conditions in the barrier facility of the Laboratory Animal Center, Central South University. All *in vivo* animal experiments were conducted in compliance with the guidelines approved by the Animal Ethics Committee of the Department of Laboratory animals, Central South University (Approval No.: APU-2026-0118).

## Declaration of competing interests

The authors declare no conflict of interest.

## Data Availability

Data will be made available on request.
