# Supplementary material for: CD-73 blocking black phosphorus nanosheets hitchhiked on erythrocytes via affinity difference of AS1411 aptamer for targeted photothermal-immunotherapy of lung cancer
Source: Mater Today Bio. 2026 Aug 13;40:103559. doi: 10.1016/j.mtbio.2026.103559 (PMC13507855; doi:10.1016/j.mtbio.2026.103559)
Supplement: Supplementary file 1 [file mmc1.docx]

**Supporting information for**

**CD-73 blocking black phosphorus nanosheets** **hitchhiked on erythrocytes *via* affinity difference of AS1411 aptamer for targeted photothermal-immunotherapy of lung cancer**

Qiao Liu^#^, Niu Liu^#^, Zhiqi Lao^#^, Jian Qin, Yuting Zhong, Kaiwei Hou, Yuetao Zhao^*^

Q. Liu, N. Liu, Y. Zhong, K. Hou, Prof. Y. Zhao

Department of Biochemistry and Molecular Biology, School of Life Sciences, Central South University, No.172 Tongzipo Road, Changsha, 410013, China

Dr. Z. Lao

Institute of Biomedicine and Biotechnology, Shenzhen Institute of Advanced Technology, Chinese Academy of Sciences, Shenzhen, China

Dr. J. Qian

Changsha Stomatological Hospital, School of Stomatology, Hunan University of Chinese Medicine, Changsha, Hunan, China

^#^ Qiao Liu, Niu Liu, an Zhiqi Lao contributed equally to this work.

^*^Correspondence:

Yuetao Zhao, E-mail: ytzhao@csu.edu.cn

**Materials and Methods**

**1. Materials**

The black phosphorus (BPs) crystal powder was provided by Hubei Xingfa Chemical Industry Group Co. LTD. 1-methyl-2-pyrrolidone (NMP) was provided by Aladdin. HEPES buffer and MnCl_2_ were provided by Solarbio and MACKLIN, respectively. The nucleic acid aptamers, including Apt, Lib, 5'-cy5-labeled Apt, and Lib, were provided by Sangon Biotech (Shanghai) Co., Ltd. HAuCl_4_, CTAB, NaBH_4_, AgNO_3_, HCl and ascorbic acid were purchased from InnoChem, SIGMA, SIGMA, Huishi, SIGMA, and Alfa Aesar, respectively. AMPCP was purchased from Sigma-Aldrich. BSA, Actin, and Nucleolin were provided by InnoChem, Shanghai Biotechnology Co., and TargetMol, respectively. The Cell Counting Kit-8 (CCK-8) and Calcein-AM/PI Assay Kit were provided by Biosharp. The Enhanced ATP Assay Kit was provided by Beyotime. The HMGB1 antibody, CRT antibody, and AF549-labeled secondary antibody were all purchased from HUABIO. BD Biosciences provided flow cytometry antibodies, including 7-AAD, CD45-APC-Cy-7, MHC II-PE, aCD11C-PE-Cy7, CD80-APC, CD86-BV421, CD8-PerCP-cy5, CD3-BV510, CD8-FITC, CD4-APC, CD25-BV421, CD11b-PE-Cy7, and Ly-6G/Ly-6C-PE. Other cell culture chemicals, including DMEM high glucose medium, penicillin-streptomycin, RPMI-1640 medium, trypsin, and fetal bovine serum (FBS), were provided by Gibco.

**2. Preparation of BPs**

BPs were prepared using the procedure described in ^[1]^. Specifically, 20 mg of BPs crystal powder was placed in an onyx mortar and thoroughly ground for 20 min. N-Methyl-2-pyrrolidone (NMP) was then added in portions to achieve a total volume of 25 mL. After probe sonication for 12 h at the power of 1200 W (ultrasonic frequency: 19-25 kHz, on/off cycle: 3 s/4 s) in an ice bath. The solution was centrifuged for 10 min at 12000 × g to remove large BPs sheets, then the aspirated supernatant was centrifuged for 30 min at 21000 × g, after which the resulting precipitate was collected as BPs. BPs were dissolved in NMP for storage.

**3. Preparation of AMPCP@Apt@BPs**

2.5 mM HEPES buffer and 6.25 mM MnCl_2_ were mixed with 20 μg BPs, after gently inverting every 30 min. After the rotation, 10 μL of Apt (100 μM) was added to the reaction system, rotary shaking at 37℃ for 1 h. Next, 26 μL of AMPCP（10 mM ） was added, and the reaction was shaken at 37 °C for 1 h. The mixture was centrifuged at 12000 × g for 5 min, discarding the supernatant and resuspending the precipitate with ddH_2_O to obtain the AMPCP@Apt@BPs.

**4. Determination of the drug loading capacity of AMPCP@Apt@BPs**

Drug loading efficiency was measured by UV-Vis-NIR spectrum. Briefly, centrifugation was performed (12000 × g, 5 min) to collect unloaded AMPCP in the supernatant and measured the absorbance at 260 nm. The amounts of unloaded AMPCP were measured according to the standard curve and corresponding solution volume. The drug loading capacity was evaluated by the following equation (Eq. (1)):

$$\text{Loading capacity(\%)=}\frac{\text{m}_{\text{1}}\text{-}\text{m}_{\text{2}}}{\text{m}_{\text{1}}}\text{×100\%}$$

m_1_ represents the initial concentration of AMPCP in the mixed system, and m_2_ represents the concentration of unloaded AMPCP in the supernatant.

**5. Preparation of AMPCP@Apt@BPs@RBC**

50 μL fresh whole blood from C57 mice was added to the anticoagulation tube. Purified red blood cells (RBC) were collected by centrifuging fresh whole blood at 800 × g for 5 min at 4℃ and washing twice with DPBS. The cy5-labeled Apt and Lib (100 µM) reacted with BPs to form cy5-AMPCP@Apt@BPs and cy5-AMPCP@Lib@BPs. Then, they were inoculated together with RBC into a 96-well plate and incubated at 37°C for 24 h. The RBC were attached with cy5-AMPCP@Apt@BPs and cy5-AMPCP@Lib@BPs, and the mixture was centrifuged at 800 × g for 5 min at 25°C to obtain AMPCP@Apt@BPs@RBC and AMPCP@Lib@BPs@RBC.

**6. Preparation of Apt@GNRs**

Apt@GNRs were prepared using the procedure described in ^[2]^. GNRs were synthesized using the seed-mediated growth approach. The preparation of the gold seed solution involves adding 2 mL of 0.2 M CTAB aqueous solution to 2 mL of 0.5 mM HAuCl_4_ solution under vigorous stirring, followed by the addition of 40 μL of 6 mM freshly prepared NaBH_4_. Before usage, the gold seed solution was allowed to rest at 37℃ for almost 2 h.The preparation of the growth solution involves the sequential mixing of 30 mL 0.2 M CTAB, 6mL 5 mM HAuCl_4_, 75 μL 0.1M AgNO_3_, 75 μL 1.2 M HCl, and 3.7 mL 10 mM ascorbic acid. After the mixture was gently rotated, the color changed from dark orange to colorless. Then, 100 μL of the gold seed solution was quickly added. The growth solution was allowed to rest at 37℃ overnight. The mixture was centrifuged at 10000 rpm for 10 min, and the precipitate was redissolved in ddH_2_O to obtain the Au NPs.

The Apt sequence (5′-cy5-GGTGGTGGTGGTTGTGGTGGTGGTGG-3′-SH) was purchased from Sangon Biotech (Shanghai) Co., Ltd. 30 μL SH-Apt (100 μM) was added to 4 nmol Apt@GNRs and stirred for 24 h. The mixture was centrifuged at room temperature to obtain Apt@GNRs.

**7. Characterization of AMPCP@Apt@BPs and AMPCP@Apt@BPs@RBC**

The TEM images were acquired on the Tecnai G2 F20 S-Twin transmission electron microscope at an acceleration voltage of 200 kV. UV-NIR absorption spectra were acquired at room temperature on a T2601 Ultraviolet-Visible Spectrophotometer (Yoke Instruments) with QS-grade quartz cuvettes. The zeta potentials and dynamic diameter were measured on a NanoBrook 90Plus analyzer (Brookhaven Instruments). The SEM images were acquired on the Hitachi S-3400N scanning electron microscope at an acceleration voltage of 10 kV.

**8. Hemolysis assay**

300 μL fresh whole blood from C57 mice was added to the anticoagulation tube. Purified RBC were collected by centrifuging fresh whole blood at 800 × g for 5 min at 4℃ and washing twice with DPBS. The red blood cells were resuspended with DBPS, H_2_O, AMPCP@Apt@BPs (60 μg/ml BPs), and AMPCP@Lib@BPs (60 μg/ml BPs), and cocultured at 37°C for 5 h. Afterwards, the mixture was centrifuged at 800 × g for 5 min. To further eliminate the influence of the nanomedicine, the supernatant was centrifuged at 12000 × g for 10 min. The released heamoglobin in the supernatant was determined by measuring the absorbance at 540 nm using a UV-Vis spectrophotometer to assess the hemolytic activity. The hemolysis rate was evaluated by the following equation (Eq. (2)):

$$\text{Hemolysis rate(\%)=}\frac{\text{Abs}_{\text{Sample}}\text{-}\text{Abs}_{\text{DPBS}}}{\text{Abs}_{\text{H}\text{2}\text{O}}\text{-}\text{Abs}_{\text{DPBS}}}\text{×100\%}$$

Abs_sample_ is the absorbance of the sample; Abs_H2O_ and AbsDPBS were used as the positive control and negative control, respectively. The hemolysis rate of the positive control was set to 100%.

**9. Complete blood count (CBC) analysis**

The levels of red blood cells, mean corpuscular volume (MCV), hematocrit (HCT), standard deviation of red blood cell volume distribution width (RDW-SD), and coefficient of variation of red blood cell volume distribution width (RDW-CV) were measured using the Sysmex XN-1000-B1.

**10. Incubation of Apt@GNRs with BSA, Actin, and Nucleolin**

Separately, BSA, Actin, and Nucleolin were added to 2 nmol Apt@GNRs. The resulting mixtures were incubated for 2 - 3 days at room temperature, after which UV-Vis-NIR absorption spectra were recorded over a wavelength range of 200 - 1100 nm using a UV-Vis spectrophotometer.

**11. *In vitro* cellular targeting**

The Apt sequence (5′-cy5-GGTGGTGGTGGTTGTGGTGGTGGTGG-3′) and Lib sequence (5-cy5-ATCCGTTACTCAGAACTAGCNNNNNNNNNNNNNNNATT CGACTCATACATGCATT-3′) were purchased from Sangon Biotech (Shanghai) Co., Ltd. cy5-labeled Apt and Lib (100 µM) were reacted with BPs to obtain Apt@BPs-cy5 and Lib@BPs-cy5, respectively. LLC cells were harvested by trypsin digestion and centrifuged at 600 rpm for 5 min. Following appropriate dilution, the cells were counted, and 1 × 10^5^ cells were taken. Whole blood (20 μL) from C57 mice was collected into an anticoagulant tube and centrifuged at 800 × g for 5 min at 4℃. The supernatant was discarded, and the red blood cells were washed twice with DPBS. Subsequently, 5 × 10^4^ LLC cells and 5 × 10^6^ RBC were separately resuspended in Apt@BPs-cy5 and Lib@BPs-cy5 solutions; the mixture was inoculated into a 96-well plate and incubated at 37℃ for 3 h. After incubation, the culture medium was removed, and the cells were washed twice with DPBS. Nuclei were stained with 50 μM Hoechst for 15 min, followed by two additional washes with DPBS. Fluorescence signals of cy5 were then observed using an inverted fluorescence microscope.

**12. *In vitro* transfer of nanomaterial from RBC to LLC cells**

cy5-labeled Apt (100 µM) was reacted with BPs to obtain Apt@BPs-cy5. Apt@BPs-cy5 was then co-seeded with RBC into a 96-well plate and incubated at 37℃ for 24 h to generate Apt@BPs@RBC-cy5. LLC cells and RBC were seeded into a 96-well plate at a ratio of 1:10 and incubated at 37℃ for 24 h. The cells were subsequently stained with 50 μM Hoechst for 15 min to label the nuclei, followed by two washes with DPBS. An inverted fluorescence microscope was used to observe whether the fluorescence signal of Apt@BPs-cy5 had transferred from RBC to LLC cells.

**13. Photothermal effect of AMPCP@Apt@BPs@RBC**

To investigate the photothermal performance, AMPCP@Apt@BPs@RBC (0, 20, 40, and 60 µg/mL) was added to a 96-well plate. The samples were then irradiated with an 808 nm near-infrared laser at a power density of 1.0 W/cm^2^ for 10 min, during which the temperature changes were recorded in real time at one-minute intervals.

To evaluate the photothermal effect of different materials, 100 µL of 60 µg/mL AMPCP@Apt@BPs, Apt@BPs@RBC, and AMPCP@Apt@BPs@RBC were added to a 96-well plate respectively. The samples were then irradiated with an 808 nm near-infrared laser at a power density of 1.0 W/cm^2^ for 10 min, and the temperature changes were recorded in real time at one-minute intervals to compare the photothermal performance of the various materials.

**14. *In vitro* cytotoxicity**

LLC, A549, and Beas-2b cells (4 × 10^4^ cells per well) were seeded into 96-well plates and incubated overnight. The cells were then co-incubated for 24 h with different concentrations of AMPCP@Apt@BPs@RBC (0, 20, 40, and 60 μg/mL). After 24 h, the culture medium was removed, and the cells were washed twice with DPBS. Subsequently, cell viability was assessed using the CCK-8 assay.

**15. *In vitro* cell ablation**

LLC and A549 cells (4 × 10^4^ cells per well) were seeded into 96-well plates and incubated overnight. The cells were then co-incubated with different concentrations of AMPCP@Apt@BPs@RBC (0, 20, 40, and 60 μg/mL) for 6 h. After 6 h, the cells were exposed to 808 nm near-infrared laser irradiation at 1.0 W/cm^2^ for 10 min to induce photothermal therapy. Following irradiation, the cells were further cultured for 12 h, after which the 96-well plates were washed twice with DPBS. Cell viability was assessed using the CCK-8 assay. The cell viability was evaluated by the following equation (Eq. (3)):

$$\text{Cell viability} (\%)=\frac{{{Abs}_{Sample}-Abs}_{DMEM}}{{Abs}_{Control-}{Abs}_{DMEM}}\times100\%$$

Abs_sample_ is the absorbance of the sample, Abs_control_ is the absorbance of control wells, and Abs_DMEM_ is the absorbance of blank wells.

**16. *In vitro* calcein AM/PI live-dead staining**

LLC and A549 cells (4 × 10^4^ cells per well) were seeded into 96-well plates and cultured overnight. The cells were then co-incubated with various concentrations of AMPCP@Apt@BPs@RBC (0, 20, 40, and 60 μg/mL) for 6 h. After 6 h, the cells were irradiated with an 808 nm near-infrared laser at 1.0 W cm^-2^ for 10 min and subsequently cultured for an additional 12 h. Following the 12 h incubation, 100 µL of Calcein AM/PI was added to each well. After a 30 min incubation at 37℃ in the dark, the cells were observed and photographed using an inverted microscope. Separately, LLC, A549, and Beas-2b cells (4 × 10^4^ cells per well) were seeded into 96-well plates and cultured overnight. The cells were then co-incubated with different concentrations of AMPCP@Apt@BPs@RBC (0, 20, 40, and 60 μg/mL) for 24 h. After 24 h, added 100 µL of Calcein AM/PI to each well. After 30 min incubation at 37℃ in the dark, the cells were observed and photographed using an inverted microscope.

**17. *In vitro* photothermal induced immunogenic cell death (ICD) of tumor cells**

LLC and A549 cells (4 × 10^4^ cells per well) were seeded into 96-well plates and cultured overnight. The cells were then divided into six groups according to the experimental protocol: DPBS, AMPCP@Apt@BPs, AMPCP@Apt@BPs@RBC, Apt@BPs@RBC + NIR, AMPCP@Apt@BPs + NIR, and AMPCP@Apt@BPs@RBC + NIR (BPs 60 μg/mL, AMPCP 0.125 μM). After co-incubation of the materials with the cells for 6 h, the photothermal treatment groups were exposed to 808 nm near-infrared laser irradiation at 1.0 W/cm^2^ for 10 min.

Detection of calreticulin (CRT): After an additional 12 h of incubation, the cells were fixed with 4% paraformaldehyde for 30-60 min, permeabilized with 0.1% Triton X-100 for 5-10 min at room temperature, and then blocked with blocking buffer for 2 h at room temperature. The cells were subsequently incubated with primary anti-CRT antibody overnight at 4℃, followed by incubation with AF549-conjugated secondary antibody for 1 h at room temperature. Finally, after staining with Hoechst for 15 min at room temperature. Fluorescence signals were observed using an inverted fluorescence microscope.

Detection of high mobility group box 1 protein (HMGB1): After an additional 24 h of incubation, the cells were fixed with 4% paraformaldehyde for 30-60 min, permeabilized with 0.1% Triton X-100 for 1 h at room temperature, and then blocked with blocking buffer for 2 h at room temperature. The cells were subsequently incubated with primary anti-HMGB1 antibody overnight at 4℃, followed by incubation with AF549-conjugated secondary antibody for 1 h at room temperature. Finally, after staining with hochest for 15 min at room temperature. Fluorescence signals were observed using an inverted fluorescence microscope.

**18. Construction of tumor model**

Specific pathogen-free (SPF) male B/C nude mice and male C57 mice used in this study were purchased from Hunan Silek Jingda Experimental Animal Co. Ltd. and housed in the biological barrier facility of the Department of Experimental Animals, Central South University. All in vivo experiments were conducted in accordance with the requirements of the Animal Ethics Approval Forms of Central South University (Approval No.: APU-2026-0118).

After shaving the axillary area, 100 μL LLC cells (4 × 10^6^ cells) in the logarithmic growth phase were subcutaneously injected into the right hind leg of the mice. The mice were housed in an SPF-grade environment. Tumor volumes were calculated using the following formula (with both length and width expressed in millimeters): Tumor volume (mm^3^) = longest diameter × (shortest diameter)^2^ × 0.5.

**19. *In vivo* targeting and accumulation assay of Apt@BPs@RBC**

Freshly prepared BPs-cy5, Apt@BPs-cy5, and Apt@BPs@RBC-cy5 were administered via tail vein injection into the tumor-bearing B/C nude mice. The distribution of fluorescence signals in the mice was monitored at specific time points using a Caliper Ivis Spectrum in vivo imaging system. At 8 h post-injection, the fluorescence signals in the heart, liver, spleen, lungs, kidneys, and tumors were observed and compared.

**20. *In vivo* antitumor study**

When the tumor volume reached 150-200 mm^3^, tumor-bearing C57 mice were randomly divided into six groups (n = 5) and treated with DPBS, AMPCP@Apt@BPs, AMPCP@Apt@BPs@RBC, AMPCP@Apt@BPs + NIR, Apt@BPs@RBC + NIR and AMPCP@Apt@BPs@RBC + NIR. The materials were administered via tail vein injection (BPs concentration: 10 μg per mouse; AMPCP concentration: 0.1 mmol per mouse), the photothermal treatment groups were exposed to 808 nm near-infrared laser irradiation at 0.65 W/cm^2^ for 10 min, and temperature changes were recorded and imaged using an infrared thermal camera. Mouse body weight and tumor volume changes were recorded every other day. After the completion of the treatment, the mice were rested for approximately one week. Whole blood samples were then collected from the tail vein of C57 mice into anticoagulant tubes for hematological analysis. Subsequently, blood was collected from the orbital sinus, and tumor tissues as well as major organs were rapidly dissected. The tumor weight was measured using an electronic balance, and the tumors were recorded and photographed.

**21. Measurement of relative ATP levels in tumor tissue**

Approximately 100-200 μL of lysis buffer was added per 20 mg of tissue, followed by homogenization using a glass homogenizer or other homogenization equipment. After lysis, the homogenate was centrifuged at 12000 × g for 5 min at 4°C, and the supernatant was collected for subsequent assays. Upon preparation of the ATP detection working solution, the relative ATP levels in the tumor tissue were determined according to the manufacturer's instructions.

**22. Flow cytometric analysis of immune cells in tumor tissues**

Tumor tissues were digested with collagenase IV and DNase I at 37℃ with shaking for 1 h. The mixture was filtered through a 70 μm cell strainer to obtain a single-cell suspension, and red blood cell lysis buffer was added for 30 s. The mixture was centrifuged at 800 × g for 5 min at 4℃, and the cells were washed three times with DPBS. Cells were blocked with mouse Fc block and subsequently incubated with fluorescence-conjugated antibodies. The following antibody panels were used: for mature DC identification, CD45-APC-Cy7, MHC II-PE, CD11C-PE-Cy7, CD80-APC, and CD86-BV421; for CD8^+^ T cell identification, CD45-APC-Cy7, CD3-BV510, and CD8-FITC; for Treg T cell identification, CD45-APC-Cy7, CD3-BV510, CD4-APC, and CD25-BV421; and for MDSC identification, CD45-APC-Cy7, CD11b-PE-Cy7, and Ly-6G/Ly-6C-PE. Flow cytometry data were acquired using a flow cytometer and analyzed with FlowJo V10 software.

**23. Immunofluorescence of tumor tissues**

Tumor tissues were fixed by immersion in 4% paraformaldehyde for 24 h, followed by paraffin embedding and sectioning. The paraffin-embedded sections were subsequently subjected to dewaxing, rehydration, antigen retrieval, blocking, antibody incubation, and TSA fluorescent dye labeling for chromogenic detection. After these treatments, the sections were counterstained with DAPI for nuclear visualization, mounted with fluorescence mounting medium, and examined under a fluorescence microscope.

**24. *In vivo* biosafety**

During the treatment period, the condition of the mice was monitored, and body weight was measured and recorded every other day. WBC, RBC, HGB, PLT, LYMPH, and MONO were analyzed using the Sysmex XN-1000-B1. The major organs, including the heart, liver, spleen, lungs, and kidneys, were fixed in 4% paraformaldehyde for 24 h, followed by sequential staining with hematoxylin and eosin (H&E), dehydration with absolute ethanol, clearing with xylene, and mounting with neutral resin. The sections were then examined under a microscope. Serum levels of ALT and CR were measured using corresponding assay kits.

**25. Statistical analysis**

Experimental results are presented as the mean ± standard deviation (n = 3 for cell experiments, n = 5 for animal experiments). Statistical comparisons between two groups were performed using a two-tailed Student's t-test. One-way analysis of variance (ANOVA) followed by Dunnett's multiple comparison test was used to assess statistically significant differences among multiple groups. Differences were considered statistically significant at the following p-value thresholds: **p* < 0.05, ***p* < 0.01, ****p* < 0.001. All statistical analyses were conducted using GraphPad Prism 8.0.1 software.

**Reference:**

[1] Xiao X, Ma Z, Li Z, Deng Y, Zhang Y, Xiang R, Zhu L, He Y, Li H, Jiang Y, Zhu Y, Xie Y, Peng H, Liu X, Wang H, Ye M, Zhao Y, Liu J. Anti-BCMA surface engineered biomimetic photothermal nanomissile enhances multiple myeloma cell apoptosis and overcomes the disturbance of NF-κB signaling in vivo. Biomaterials. 2023 ,297:122096.

[2] Chang R, Li T, Fu Y, Chen Z, He Y, Sun X, Deng Y, Zhong Y, Xie Z,Yang Y, Liu J, Chen X, Liu H, Zhao Y, A PD-L1 targeting nanotheranostic for effective photoacoustic imaging guided

photothermal-immunotherapy of tumor. J. Mater. Chem. B. 2023, 11, 8492.

**Figure S1: (**a) AFM image of the BPs. (b) Height profiles along the white lines in Figure S1a.

**Figure S2:** DLS particle size distribution of BPs, Apt@BPs, AMPCP@BPs, and AMPCP@Apt@BPs.

**Figure S3:** Drug loading capacity of AMPCP@Apt@BPs. (a) Standard curve of the Apt. (b) Standard curve of AMPCP. (c) Absorption spectra of the supernatant.

**Figure S4:** Long-term storage stability of AMPCP@Apt@BPs. (a) Zeta potential, (b) Diameter detected by DLS, and (c) Absorption of drug released in the supernatant of AMPCP@Apt@BPs stored at -20 ^o^C at different times. (c) Zeta potential, (d) Diameter detected by DLS, and (e) Absorption of drug released in the supernatant of AMPCP@Apt@BPs stored at 4 ^o^C at different times.

**Figure S5:** (a) Schematic illustration of the preparation, and (b) TEM of Apt@GNRs.

**Figure S6**. UV-Vis-NIR absorption spectra of GNRs after co-incubation with various concentrations of (a) BSA, (b) actin, (c) nucleolin.

**Figure S7:** UV-Vis-NIR absorption spectra of Apt@GNRs after incubation with various concentrations of coagulation factor

**Figure S8.** Quantitative fluorescence analysis of LLC cells and RBC after 3 h of co-incubation with cy5-AMPCP@Apt@BPs and cy5-AMPCP@Lib@BPs.

**Figure S9.** Quantitative fluorescence analysis of LLC cells alone and of LLC cells after 0 h and 24 h co-incubation with cy5-AMPCP@Apt@BPs@RBC.

**Figure S10:** (a) Fluorescence image, and (b) corresponding quantitative fluorescence analysis of LLC cells co-incubated with cy5-labled Apt@BPs@RBC after 24 h. Blue: Hoechst; Red: cy5.

**Figure S11.** Calcein-AM/PI staining images of Beas-2b cells after 24 h of incubation with various concentrations of AMPCP@Apt@BPs@RBC. Green: Calcein-AM, n = 3.

**Figure S12.** *In vitro* photothermal ablation effect of AMPCP@Apt@BPs@RBC on a) LLC, and b) A549 cells assessed by Calcein-AM/PI staining. Green: Calcein-AM; red: PI, n = 3.

 **Figure S13:** *In vitro* photothermal ablation effect of AMPCP@Apt@BPs@RBC on B16F10 cells assessed by Calcein-AM/PI staining. Green: Calcein-AM; red: PI, n = 3.

**Figure S14:** ELISA test of HMGB1 of LLC cells under different treatment. n=3

**Figure S15.** CRT expression on the A549 cell membrane and corresponding quantitative fluorescence analysis. Blue: Hoechst; red: AF549, n = 3.

**Figure S16**. HMGB1 release from the nucleus of A549 cells and corresponding quantitative fluorescence analysis. Blue: Hoechst; Green: AF488, n = 3.

**Figure S17:** (a) Flow cytometric detection of DC maturation induction under different treatment. (b) Gating strategy of CD80^+^ CD86^+^ DCs corresponding to Figure S17a.

**Figure S18**. (a) *In vivo* imaging of tumor-bearing nude mice at 72 and 96 h following intravenous injection of nanomaterials. Red: cy5. (b) *Ex vivo* biodistribution, (c) Quantitative fluorescence analysis of nanomaterials in tumor and major organs 96 h post-injection. Red: cy5.

**Figure S19**. Quantitative fluorescence analysis of frozen tumor sections obtained from tumor-bearing nude mice at 96 h post-tail vein injection.

**Figure S20:** (a) *In vivo* imaging of tumor-bearing C57 mice at different time points after intravenous injection of nanomaterials. Red: cy5. (b) *Ex vivo* biodistribution of nanomaterials in tumors and major organs 8 h post-injection. Red: cy5.

**Figure S21.** Relative ATP levels in tumor tissue. (G1) DPBS, (G2) AMPCP@Apt@BPs, (G3) AMPCP@Apt@BPs@RBC, (G4) AMPCP@Apt@BPs + NIR, (G5) Apt@BPs@RBC + NIR, and (G6) AMPCP@Apt@BPs@RBC + NIR.

**Figure S22:** Adenosine levels in LLC cells under different treatment. (G1) DPBS, (G2) AMPCP@Apt@BPs, (G3) AMPCP@Apt@BPs@RBC, (G4) AMPCP@Apt@BPs + NIR, (G5) Apt@BPs@RBC + NIR, and (G6) AMPCP@Apt@BPs@RBC + NIR. n=4.

**Figure S23.** Flow cytometric gating strategy of CD80^+^ CD86^+^ DCs in LLC model corresponding to Figure 6a.

**Figure S24.** Quantitative flow cytometric analysis of (a) CD80⁺ CD86⁺ DCs, (b) CD8⁺ T cells, (c) Tregs cells, (d) MDSCs. (G1) DPBS, (G2) AMPCP@Apt@BPs, (G3) AMPCP@Apt@BPs@RBC, (G4) AMPCP@Apt@BPs + NIR, (G5) Apt@BPs@RBC + NIR, and (G6) AMPCP@Apt@BPs@RBC + NIR.

**Figure S25.** Flow cytometric gating strategy of CD8^+^ T and Tregs in the LLC model corresponding to Figs. 6b and 6c.

**Figure S26.** Flow cytometric gating strategy of MDSCs in the LLC model corresponding to Fig. 6d.

**Figure S27**. Quantitative immunofluorescence analysis of (a) CD8⁺ T cells, (b) CD86⁺ DCs, (c) CD11b⁺ MDSCs. (G1) DPBS, (G2) AMPCP@Apt@BPs, (G3) AMPCP@Apt@BPs@RBC, (G4) AMPCP@Apt@BPs + NIR, (G5) Apt@BPs@RBC + NIR, and (G6) AMPCP@Apt@BPs@RBC + NIR.

**Figure S28:** (a) Captured image, and (b) corresponding size analysis of spleen of mice at the endpoint of treatment from groups. (G1) DPBS, (G2) AMPCP@Apt@BPs, (G3) AMPCP@Apt@BPs@RBC, (G4) AMPCP@Apt@BPs + NIR, (G5) Apt@BPs@RBC + NIR, and (G6) AMPCP@Apt@BPs@RBC + NIR.

**Figure S29:** H&E staining of skin surrounding the irradiating point after (a) once, and (b) three times of irradiation. H&E staining of muscle surrounding the irradiating point after (c) once, and (d) three times of irradiation.

**Figure S30.** (a) AST, (b) BUN, and (c) UA level in peripheral blood. (G1) DPBS, (G2) AMPCP@Apt@BPs, (G3) AMPCP@Apt@BPs@RBC, (G4) AMPCP@Apt@BPs + NIR, (G5) Apt@BPs@RBC + NIR, and (G6) AMPCP@Apt@BPs@RBC + NIR. n=5.
